# Graviola (*Annona muricata*) Exerts Anti-Proliferative, Anti-Clonogenic and Pro-Apoptotic Effects in Human Non-Melanoma Skin Cancer UW-BCC1 and A431 Cells In Vitro: Involvement of Hedgehog Signaling

**DOI:** 10.3390/ijms19061791

**Published:** 2018-06-16

**Authors:** Jean Christopher Chamcheu, Islam Rady, Roxane-Cherille N. Chamcheu, Abu Bakar Siddique, Melissa B. Bloch, Sergette Banang Mbeumi, Abiola S. Babatunde, Mohammad B. Uddin, Felicite K. Noubissi, Peter W. Jurutka, Yong-Yu Liu, Vladimir S. Spiegelman, G. Kerr Whitfield, Khalid A. El Sayed

**Affiliations:** 1 Department of Basic Pharmaceutical Sciences, School of Pharmacy, College of Health and Pharmaceutic Sciences, University of Louisiana at Monroe, Monroe, LA 71209-0497, USA; siddiqab@warhawks.ulm.edu (A.B.S.); blochmb@warhawks.ulm.edu (M.B.B.); burhan1129@gmail.com (M.B.U.); yliu@ulm.edu (Y.-Y.L.); elsayed@ulm.edu (K.A.E.S.); 2 Department of Dermatology, School of Medicine and Public Health, University of Wisconsin, Madison, WI 53706, USA; iabdelaal@wisc.edu (I.R.); roxanechamcheu@gmail.com (R.-C.N.C.); asbabatunde@unilorin.edu.ng (A.S.B.); 3 Madison West High School, 30 Ash St, Madison, WI 53726, USA; 4 Division for Research and Innovation, POHOFI Inc., P.O. Box 44067, Madison, WI 53744, USA; sbmbeumi@pohofi.org; 5 Department of Biology, Jackson State University, Jackson, MS 39217, USA; felicite.noubissi_kamdem@jsums.edu; 6 School of Mathematical and Natural Sciences, Arizona State University, Phoenix, AZ 85306, USA; peter.jurutka@asu.edu; 7 Division of Pediatric Hematology/Oncology, Department of Pediatrics, Pennsylvania State University, College of Medicine, Hershey, PA 17033, USA; vspiegelman@pennstatehealth.psu.edu; 8 Department of Basic Medical Sciences, University of Arizona College of Medicine-Phoenix, Phoenix, AZ 85004, USA; gkw@email.arizona.edu

**Keywords:** *Annona muricata*, apoptosis, basal cell carcinoma, cutaneous squamous cell carcinoma, graviola, Hedgehog signaling pathway, natural products chemistry, non-melanoma skin cancer

## Abstract

Non-melanoma skin cancers (NMSCs) are the leading cause of skin cancer-related morbidity and mortality. Effective strategies are needed to control NMSC occurrence and progression. Non-toxic, plant-derived extracts have been shown to exert multiple anti-cancer effects. Graviola (*Annona muricata*), a tropical fruit-bearing plant, has been used in traditional medicine against multiple human diseases including cancer. The current study investigated the effects of graviola leaf and stem extract (GLSE) and its solvent-extracted fractions on two human NMSC cell lines, UW-BCC1 and A431. GLSE was found to: (i) dose-dependently suppress UW-BCC1 and A431 cell growth, motility, wound closure, and clonogenicity; (ii) induce G_0_/G_1_ cell cycle arrest by downregulating cyclin/cdk factors while upregulating cdk inhibitors, and (iii) induce apoptosis as evidenced by cleavage of caspases-3, -8 and PARP. Further, GLSE suppressed levels of activated hedgehog (Hh) pathway components Smo, Gli 1/2, and Shh while inducing SuFu. GLSE also decreased the expression of pro-apoptotic protein Bax while decreasing the expression of the anti-apoptotic protein Bcl-2. We determined that these activities were concentrated in an acetogenin/alkaloid-rich dichloromethane subfraction of GLSE. Our data identify graviola extracts and their constituents as promising sources for new chemopreventive and therapeutic agent(s) to be further developed for the control of NMSCs.

## 1. Introduction

Non-melanoma skin cancer (NMSC), the most prevalent form of cancer worldwide, is classified into two major forms, basal cell carcinoma (BCC) and cutaneous squamous cell carcinoma (cSCC) [1]. BCC, which arises in the basal cells layer of the epidermis [2,3], is the most common form of skin cancer [4], and constitutes up 80% of skin cancers and nearly a third of all cancers diagnosed in the U.S. [5,6,7,8]. SCC arises in the squamous epidermal layer, and is the second most common form of NMSC, comprising about 16% of all skin cancers [2,9,10]. Although BCCs are not usually life threatening [11], they can often cause local ulcerations, loss of function, and disfiguration if left untreated [12,13]. In contrast, cSCCs are much more dangerous due to their likelihood to invade, bleed and metastasize, and represent a major cause of morbidity and mortality worldwide [10,14,15]. Together, these forms of NMSC present a major public health burden across the world [16]; in the U.S., their incidence has increased over 300% since 1994 [17] with about 5.4 million cases being treated for NMSC yearly [9], making it the fifth highest total for all cancers [18]. The estimated total annual NMSC-related expenditure in the U.S. is $4.8 billion [5], with 80% of newly diagnosed cases occurring in adults over 60 [17], although recent increases have also been reported among younger individuals in many regions of the world [9]. Skin carcinogenesis primarily occurs on sun-exposed areas including the face, ears, head, neck, hands, scalp, etc. [13,19], and fair-skinned individuals with history of sun tanning or living near the equator [7,8,13,20,21].

The molecular basis of these NMSCs is not well understood, but it has been shown that UV exposure can initiate tumorigenesis via the induction of pro-survival pathways, counteracting apoptosis, and allowing damaged keratinocytes to survive [22]. One way this appears to happen is via aberrant regulation of the hedgehog signaling pathway (Hh), which consists of a family of secreted proteins regulating embryonic development and maintaining homeostasis in adult tissues [12]. The Hh pathway, like many dysregulated pro-survival pathways, promotes tumorigenesis through increased cell cycle progression and loss of regulation of proliferation, and is also a key target in cancer therapeutics [23]. Activating mutations of Smoothened (Smo) or suppressing mutations of Patched 1 (Ptch1) constitutively activate the Hh signaling in BCC, which is also a hallmark of sporadic BCC [24].

Current treatments for NMSC patients are predominantly surgical removal and/or radiation therapy [12,25], either of which can lead to considerable morbidities and other cosmetic consequences on mostly visible areas. Therefore, there is an urgent need to develop novel, cost-effective chemoprevention and therapeutic strategies with minimal cosmetic damage as an alternative to existing NMSC remedies. One such promising strategy is to identify and develop novel natural nutraceuticals that can specifically target cancerous cells with minimal side-effects and cosmetic damage as well as to understand their complex mechanisms of action.

**Figure 1:**
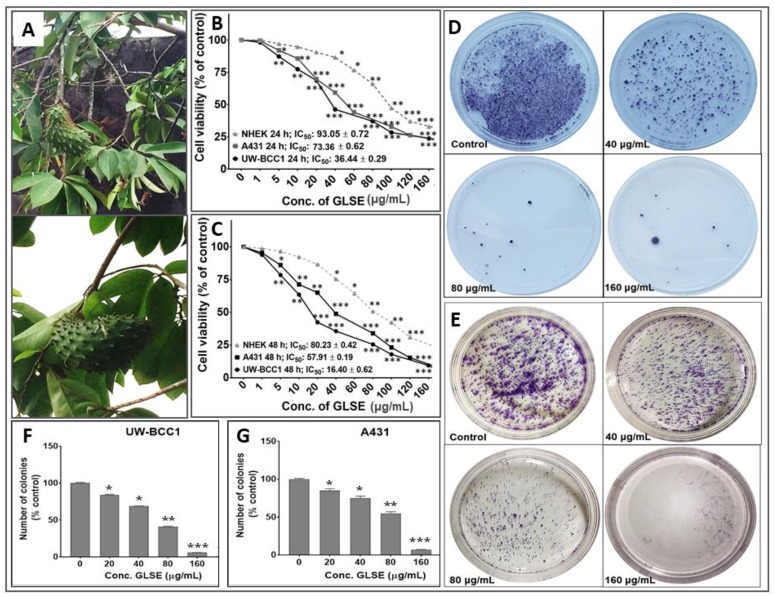
(**A**) Graviola aerial parts including leaves, stems and fruits. Effects of GLSE on UW-BCC1 and A431 cell viability after (**B**) 24 h or (**C**) 48 h and colony formation of non-melanoma skin cancer (NMSC) cells. Cells were incubated with the indicated concentration of GLSE, and percentage cell viabilities, determined by CCK-8 assay for UW-BCC1 cells, and by MTT assay for A431 and NHEK cells, were plotted against the doses of GLSE (μg/mL). Values used for plotting are means of experiments performed three times, with each concentration tested in 7–8 wells. Effects of GLSE on clonogenicity of UW-BCC1 (**D** and **F**) and A431 (**E** and **G**) cells as detected by colony formation assay. The purple color shows the density of stained cell colonies in the different treatment groups. Means for each cell line were compared against NHEKs in viability studies. Statistical differences from control cultures are shown as bar graphs with error bars representing the means ± SD in panels (**F**) and (**G**); * *p* < 0.05 and ** *p* < 0.01 and *** *p* < 0.001 vs. control (DMSO-treated) cells.

Over 47% of current anticancer drugs on the market are natural products, their derivatives or natural product synthetic mimics [26,27], and more than 25,000 identified phytochemicals have been shown to possess potent anticancer activities [14,26]. Graviola *(Annona muricata)* is a small deciduous tree of the Annonaceae family, widely distributed in tropical countries (Figure 1A and Figure S1A,B), and commonly referred to as guanabana, soursop, or Brazilian paw-paw [28,29]. Graviola is an example of a natural plant source of anti-cancer phytochemicals, and decoctions of its bark, roots, seeds, leaves, pericarp, and fruits, have been used in traditional medicine to treat ailments including diabetes, cough, skin diseases, cancers and other disorders [28,29,30], with over 212 phytochemicals identified in diverse graviola extracts [28,29,30].

Different classes of constituent “annonaceous” metabolites such as acetogenins are believed to play a major role in the anti-cancer properties of graviola on mammalian cells, in addition to many other constituents such as alkaloids, flavonoids, sterols and others [28,29,30,31]. Studies to date, all in non-skin tumor lines, suggest that the effects of graviola are selective for inhibiting the growth of cancerous cells, with minimal effects on normal cells [31,32].

The present study investigated the effects of a powdered extract of graviola aerial parts (herein referred to as GLSE), and successively extracted subfractions thereof, on two NMSC cell lines, namely UW-BCC1, derived from a basal cell carcinoma [13], and A431 [33], representing squamous cell carcinoma compared to control keratinocytes. These cell lines were chosen for their ability to form subcutaneous tumors in nude mice that resemble human non-melanoma skin cancers, and, in the case of A431, a long history of use as a cell line with squamous cell carcinoma-like properties. Our results demonstrate for the first time that GLSE is able to inhibit the growth and viability of both BCC and SCC cell lines while also exerting an inhibitory effect on Hh signaling in vitro. Preliminary analysis of solvent subfractions of graviola powder reveals that the anti-cancer activities are concentrated mainly in the acetogenin- and alkaloid-rich dichloromethane (DCM) fraction.

## 2. Results

### 2.1. GLSE Inhibits Cell Proliferation, Viability and Clonogenicity of UW-BCC1 and A431 Cell Lines

Since different parts of the graviola plant have been reported to possess anti-cancer activities against multiple non-skin cancer cell types, we first investigated the effect of GLSE on the growth, viability, migration and clonogenic potential of UW-BCC1 and A431 cell lines as compared to control non-cancerous human epidermal keratinocytes (NHEKs). Employing the 3-(4-dimethylthiazol-2-yl)-2,5-diphenyltetrazolium bromide (MTT), trypan blue dye exclusion and Cell Counting Kit-8 (WST/CCK-8) assays, we observed that GLSE exerted significant time- and dose-dependent inhibition of cell growth in both UW-BCC1 and A431 cell lines after 24 and 48 h to a greater extent than in control NHEKs (Figure 1B,C). Time course analysis revealed that most differences between cancer vs. control cells were already evident at 24 h, with only modestly greater effects at 48 h, indicating that the response to GLSE treatment occurs within 24 h. We also observed that GLSE elicited distinctive responses vis-a-vis the two different cell lines, with UW-BCC1 cells being responsive at IC_50_ values (36.44 μg/mL and 16.40 μg/mL), compared to A431 cells (IC_50_ values of 73.36 μg/mL and 57.91 μg/mL) for 24 and 48 h respectively (see Figure 1B,C and Figure S1C). By comparison, inhibition of cell growth and proliferation of NHEKs by treatment with GLSE required higher doses (IC_50_ values of 93.05 μg/mL and 80.23 μg/mL for 24 and 48 h, respectively) (See Figure 1B,C and Figure S1C). Notably, the doses of GLSE required to achieve an equivalent inhibition of cell viability in UW-BCC1 are over 3.5-fold less than those of A431, and 5.2-fold less than that of the normal epithelial cells, NHEK, especially in the range of doses between 5–80 µg. In turn, the A431 corresponding doses were approximately 1.5-fold less than that of NHEK. These results led us to focus our interpretations of later experiments on the dose range in which the effect differential between non-cancerous vs. cancerous cells was maximized, namely between 5–80 µg/mL.

One caveat to the above interpretation is that a different assay was used for UW-BCC1 cells (WST/CCK-8) than for A431 and NHEK cells for the results shown in Figure 1B,C. We therefore assessed the effect of GLSE (0–160 µg/mL) also on colony formation of UW-BCC1 and A431 cells after 14–16 days. We observed a significant and dose-dependent inhibition of colony formation with UW-BCC1 (Figure 1D,F) as well as with A431 (Figure 1E,G) cells relative to untreated controls. We detect a slightly greater sensitivity of the UW-BCC1 line to GLSE as compared to A431 (see Figure 1F,G, compare 40 and 80 µg/mL doses). In a third type of cell viability assay (trypan blue), UW-BCC1 once again appears to be slightly more sensitive (see Figure S1D,E—compare % viabilities at the 40 µg/mL dose).

### 2.2. GLSE Suppresses Transwell Membrane Migration and Scratch Wound Healing

The inhibitory potential of GLSE on UW-BCC1 and A431 cell migration across a trans-well membrane and on scratch wound closure was assessed, and as shown in Figure S2A–C, all tested doses of GLSE significantly and dose-dependently inhibited UW-BCC1 and A431 cell migration across trans-well membrane (Figure S2A–C). This effect on migration is likely due to an effect on cell viability (cytotoxicity) as shown in Figure 1C at the corresponding 48 time point.

In addition, a dose-dependent inhibition of UW-BCC1 and A431 wound closure by GLSE was observed (Figure S3). Again, cytotoxicity is likely to be a major factor in the inability of the UW-BCC1 and A431 cells to close the wound during the 30 h post-scratch incubation period. Regardless of the mechanism, these effects were observed at doses as low as 15–30 µg/mL, a range in which GLSE effects on cancer cells are markedly greater than effects on non-cancerous cells (Figure 1B,C).

### 2.3. GLSE Induces G0/G1-Phase Cell Cycle Arrest in UW-BCC1 and A431 Cell Lines

**Figure 2:**
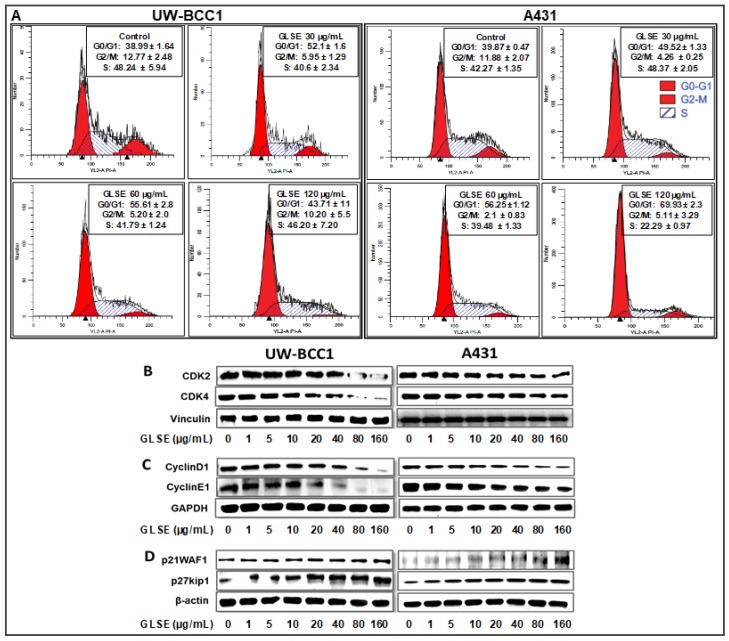
GLSE induces G_0_/G1 phase cell cycle arrest of non-melanoma skin cancer cells. UW-BCC1 and A431 cells treated with GLSE for 24 h were stained with the Apo-Direct kit following the manufacturer’s protocol and analyzed by flow cytometry. Plots and percentage distribution of cell population in the G_0_/G_1_, G_2_/M and S phases of the cell cycle are shown in the insert in each panel: (**A**) results from UW-BCC1 cells at different GLSE doses, and (**B**) results from A431 cells. (**B**–**D**) Quantification of effects of GLSE treatment on cell cycle regulatory proteins. Whole cell lysates of UW-BCC1 (bottom left set of images in panels (**B**)–(**D**)) and A431 (bottom right set of images) with/without GLSE (0–160 µg/mL: 24 h) were subjected to SDS-polyacrylamide gel electrophoresis. Blots containing resolved proteins from UW-BCC1 and A431 cells were analyzed by immunoblotting with antibodies for CDK2, CDK4, Cyclin D1, Cyclin E1, p21WAF1 or p27kip1. Equal loading was confirmed by re-probing with β-Actin, GAPDH or vinculin as loading controls. The immunoblot images shown are representative of three independent experiments with similar results. Quantification data are shown in the supplementary Figure S4.

The effects of GLSE (three doses) on cell cycle distribution and apoptosis were examined by flow cytometry using the APO-Direct kit, which can concurrently analyze both cell cycle distribution and apoptosis. DNA cell cycle profile analysis performed in linear growth phase revealed significant dose-dependent increases in the number of cells in the G_0_/G_1_ phase of the cell cycle in both cell lines compared to no-treatment controls. The G_0_/G_1_ phase distribution of UW-BCC1 cells after treatment with GLSE (0, 30, 60 and 120 µg/mL) was 38.99%, 52.1% and 55.61%, 43.7%, respectively (Figure 2A, top left four panels). For A431, the corresponding values were 39.87%, 49.52%, 56.25%, and 69.93% (Figure 2A; top right four panels). The increases in the G_0_/G_1_ phase cell population were accompanied by decreases in the G_2_/M and S phase cell populations of both cell lines except for an observed decline at the 120 µg/mL dose of GLSE in the UW-BCC1 cells. The declines in all phases at this highest dose of GLSE (120 µg/mL), especially in the UW-BCC1 cell line, are suggestive as evidence of toxicity considering that the experiments were extensively repeated (therefore these data should be interpreted with caution). The cell cycle distribution of the DMSO vehicle-treated cells was found to be similar to the control untreated cells; hence, we present only DMSO treated data as “Controls” in Figure 2A. The relatively low G_0_/G_1_-phase population of DMSO treated cells was possibly because growing, non-synchronized cells were used in these experiments.

Since our studies demonstrated that GLSE treatment of UW-BCC1 and A431 cells results in a G_0_/G_1_-phase arrest, we next examined by immunoblot analysis the effect of GLSE on cell cycle regulatory molecules that are operative in the G_1_ phase of the cell cycle. Thus, we assessed the effect of GLSE on the expression of p21WAF1, known to partly regulate entry at the G_1_-S-phase transition checkpoint as well as to induce apoptosis [34]. Our results revealed a dose-dependent induction of p21WAF1 and of its partner protein p27kip1 in both UW-BCC1 (Figure 2D, bottom left panels) and A431 (Figure 2D, bottom right panels) cells compared with the basal levels in controls (see the supplementary Figure S4 for quantitation). Additionally, the effect of GLSE on the protein expression of cyclin dependent kinase (CDKs) as well as cyclins showed a dose-dependent decrease in expression of CDK2, and CDK4 (Figure 2B) as well as in cyclins D1 and E1 (Figure 2C) in both UW-BCC1 (left panels) and A431 (right panels) (again, see the supplementary Figure S4 for quantitation). We conclude from these data that GLSE-induced cell cycle arrest is likely mediated via an induction of p21WAF1 and p27kip1, with a concomitant inhibition of CDK2 and CDK4, along with cyclins D1 and E1.

### 2.4. GLSE Induces Apoptosis in UW-BCC1 and A431 Cell Lines

**Figure 3:**
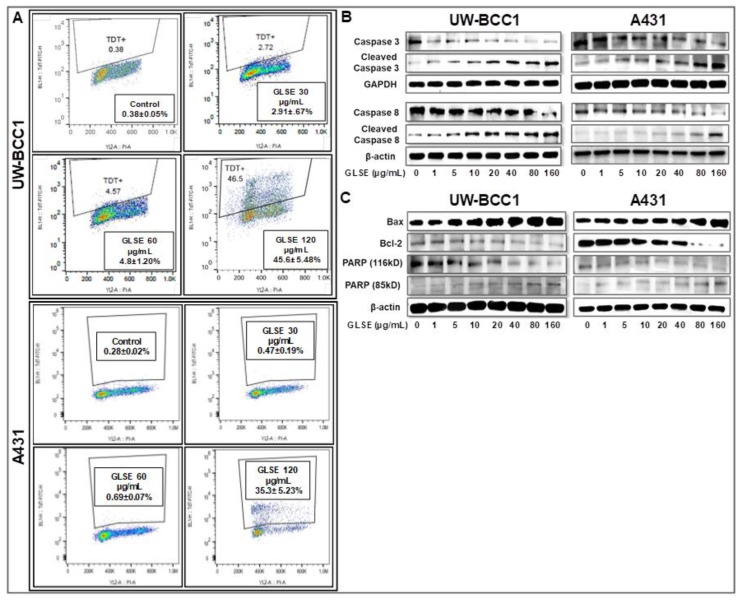
GLSE induces apoptosis of UW-BCC1 and A431 cells through activation of caspases 3/8, and PARP, and suppression of Bcl-2. (**A**) UW-BCC1 and A431 cells treated with or without GLSE (0–120 µg/mL: 24 h) were labeled with the Apo-Direct kit and analyzed by flow cytometry. Percentage of apoptotic cells observed (mean ± SD) with each dose of GLSE are shown in the box inserts in each panel. All experiments were performed in triplicate. (**B**,**C**) Whole cell lysates of UW-BCC1 (left panels) and A431 cells (right panels) treated with/without GLSE (0–160 µg/mL, 24 h) were subjected to SDS-polyacrylamide gel electrophoresis and blots were probed with antisera to proteins involved in apoptosis pathways, showing (**B**) expression levels of caspase-3 and caspase-8 in both the intact and cleaved forms; and (**C**) expression levels of Bax, Bcl-2 and PARP, the latter in both the 116 kDa and 85 kDa forms. Equal protein loading was confirmed by re-probing with β-Actin or GAPDH. The immunoblots shown are representative of three independent experiments with similar results. Data represent the means of three independent experiments each conducted in triplicates ± SD vs. control (DMSO-treated cells), and bar graphs for (**B**) and (**C**) representing the means ± SD are presented in Figure S6.

To assess whether GLSE-induced growth inhibition of the NMSC cells comprises induction of apoptosis, we employed the APO-Direct kit along with flow cytometry and Western blotting to evaluate the expression of apoptotic markers. GLSE treatment showed a significant dose-dependent increase in the population of apoptotic in UW-BCC1 cells compared with vehicle-treated controls (Figure 3A; top four panels) and A431 cells (Figure 3A; bottom four panels), reaching a maximum at 120 μg/mL of GLSE. The relatively high percentage of apoptotic cells at this highest dose, especially in the UW-BCC1, is consistent with the dramatic loss of cells and lack of cell cycle resolution in the results for the same cell line and GLSE dose in Figure 2A.

Next, to confirm whether the observed decrease in cell viability is linked to induction of apoptosis, we performed caspase-3/7 activity assays (Figure S5) and validated the results with immunoblot analyses of caspases and other proteins involved in cellular apoptosis (Figure 3B,C). Caspase-3, a member of the caspase family of aspartate-specific cysteine proteases, plays a central role in the execution of the apoptotic program. Using a caspase-3/7 activity assay, we observed a dose-dependent increase in caspase-3/7 activity in both UW-BCC1 cells (Figure S5A) and A431 cells (Figure S5B). By Western blotting, we found that GLSE-treated UW-BCC1 and A431 cells showed a dose-dependent increase in the expression of activated/cleaved Caspase-3 and Caspase-8 (Figure 3B), indicating that the apoptosis pathway is a major mechanism of GLSE-induced cell death. PARP is one of several known cellular substrates of caspases and cleavage of PARP by caspases is considered a hallmark of apoptosis [35]. As shown in Figure 3C (bottom panels), GLSE treatment of UW-BCC1 and A431 cells also resulted in a dose-dependent cleavage of PARP from its 116 kDa to its 85 kDa form, which is also indicative of the induction of both the intrinsic and extrinsic pathways of apoptosis (see Figure S6 for quantitation of these results). Furthermore, the Bcl-2 family of proteins (Figure S6), also involved in apoptosis, were quantified, and we observed that both cancer cell types showed a dose-dependent increase in the expression of pro-apoptotic Bax, and a concomitant decrease in anti-apoptotic Bcl-2 protein, leading to a dramatic increase in the Bax/Bcl-2 ratio (Western blots in Figure 3C and quantitation in Figure S6).

### 2.5. GLSE Modulates the Hedgehog Signaling Pathway Components in UW-BCC1 and A431 Cell Lines

**Figure 4:**
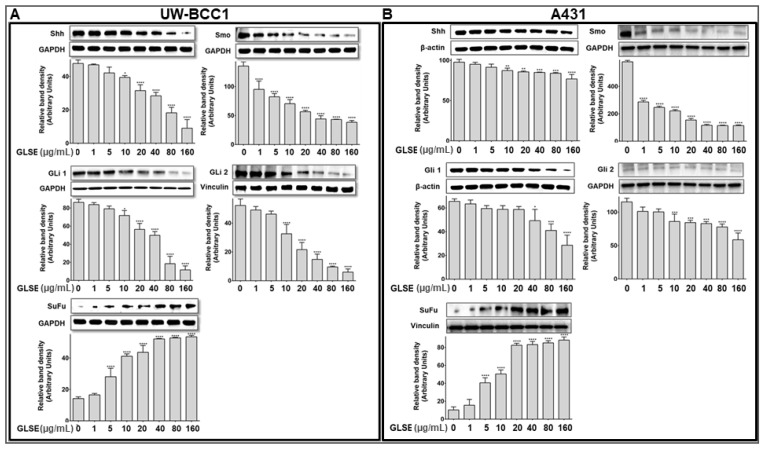
GLSE modulates Hedgehog Signaling Pathway Components in UW-BCC1 and A431 Cells. Whole cell lysates of (**A**) UW-BCC1 and (**B**) A431cells treated with/without GLSE (0–160 µg/mL: 24 h) were subjected to SDS-polyacrylamide gel electrophoresis and blots were probed with antisera to hedgehog pathway proteins Shh, Smo, Gli1, Gli2, and SuFu. Equal loading was confirmed by re-probing with GAPDH, β-Actin, GAPDH and vinculin. The immunoblots shown are representative of three independent experiments, each conducted in duplicate, which all gave similar results. Bars represent the means ± SD. * *p* < 0.05, ** *p* < 0.01, *** *p* < 0.001 and **** *p* <0.0001 vs. control (DMSO-treated) cells.

We previously demonstrated that the hedgehog (Hh) and Wnt signaling pathways are dysregulated in UW-BCC1 cells [11]. In the current study, we utilized immunoblotting to analyze several components of the Hh pathway including Smo, Gli 1/2, Shh and SuFu, and observed that GLSE treatment dose-dependently decreased the protein expression of Shh and Smo as well as Gli 1 and Gli 2 in both UW-BCC1 and A431 cells (Figure 4A,B). This inhibition was associated with a simultaneous dose-dependent increase in the expression of the negative regulator, SuFu, in both UW-BCC1 and A431 (Figure 4A,B, bottom panels). Many of these effects occurred at concentrations less than 40 µg/mL, with GLSE reaching its maximum effect on Smo at that concentration. Taken together, these data provide evidence that the Hh pathway is blunted upon treatment with GLSE, thus inhibiting Hh-dependent neoplastic growth at relatively low concentrations of GLSE in these two cancer cell lines.

### 2.6. Extraction of Graviola Aerial Parts Powder with Hexane, Dichloromethane or Methane Yields Fractions with Distinct Abilities to Inhibit UW-BCC1 and A431 Cell Viability

Fractionation of extract for identification of active ingredient-enriched components was performed by successive extraction of graviola stem and leaf powder with solvents of increasing polarity including *n*-hexane (least polar), dichloromethane (DCM, medium polarity), and methanol (MeOH, highest polarity). Each of these solvents is expected to extract graviola ingredients of differing polarities, and the extracts were then investigated for their growth inhibitory effects on UW-BCC1, A431 and a normal primary foreskin epidermal keratinocyte (NHEKn) cells using an MTT assay as described in Methods.

**Figure 5:**
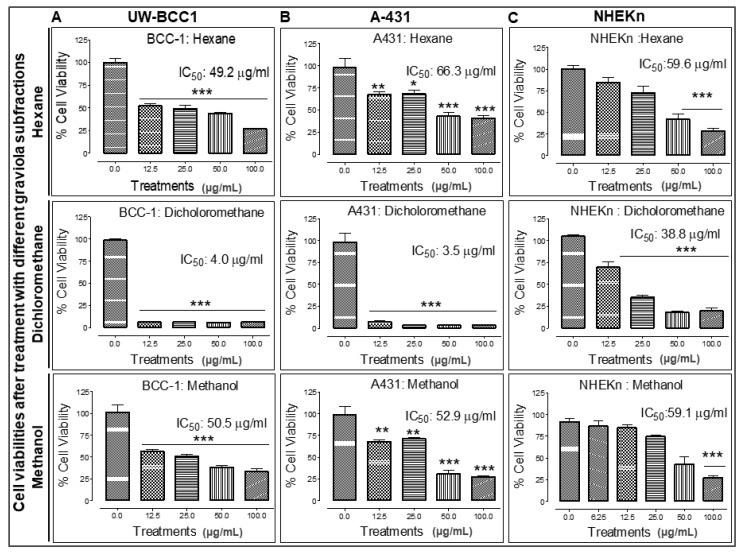
Different solvent-extracted fractions of graviola stem and leaf powder extract display differential inhibition of cell viability in non-melanoma skin cancer (NMSC) cells. UW-BCC1 and A431 as well as control NHEKn cells were treated with one of three fractions of graviola (*n*-hexane, dichloromethane, or methanol) for 48 h, and cell viability was determined by MTT assay. Bar graphs show the effect (Mean ± SD) of each fraction on the % viability after each treatment, with IC_50_ values in (**A**) UW-BCC1, (**B**) A431 and (**C**) NHEKn cells, at 48 h, shown above the bars. All experiments were performed in triplicate. Details are described in Methods. The p values vs. control (DMSO-treated) cells: * *p* < 0.05 and ** *p* < 0.01 and *** *p* < 0.001.

All solvent extracted fractions caused significant dose-dependent decreases in both cell UW-BCC1 and A431 cell viability. However, the DCM fraction proved to be by far the most potent in inhibiting the proliferation of both UW-BCC1 (IC_50_ of 4.0 µg/mL) and A431 cells (IC_50_ of 3.5 µg/mL) cells. Importantly, the IC_50_ values for the DCM fraction in both cell lines were 10-fold higher than in noncancerous NHEKn cells (IC_50_ of 38.8 µg/mL) after 48 h of treatment compared to the two NMSC lines (Figure 5, panels A–C). The other two extracts showed lower potencies: the hexane extract yielded an IC_50_ of 49.2 µg/mL with UW-BCC1 cells, an IC_50_ of 66.3 µg/mL with A431, and an IC_50_ of 59.6 µg/mL with NHEKn; and the MeOH extract inhibited UW-BCC1 viability with an IC_50_ of 50.5 µg/mL, A431 with an IC_50_ of 52.9 µg/mL and NHEKn with an IC_50_ of 59.1 µg/mL, respectively (Figure 5A–C). Furthermore, as shown in Supplementary Figure S7, all cells treated with these solvent extracts displayed characteristic dose-dependent morphological changes consistent with apoptotic cell death.

To confirm whether the observed decreases in cell viability and morphological changes were linked to the induction of apoptosis, we next determined the effect of the most potent graviola fraction, DCM on induction of apoptosis in cells using Annexin V tagged to FITC/Propidium iodide (PI) staining 48 h after treatment. We were able to confirm that the cells treated with the DCM fraction showed increased green/red fluorescence staining in contrast to untreated controls, indicating that this treatment induced apoptosis in both the UW-BCC1 and A431 skin cancer cell lines (Figure S8A,B).

### 2.7. Chemical Characterization of Different Solvent-Extracted Fractions of Graviola Leaf and Stem Powder

**Figure 6:**
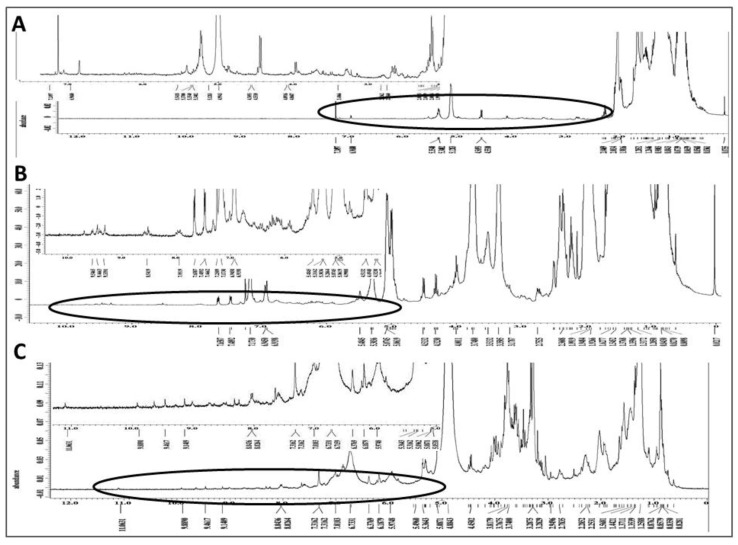
Phytochemical fingerprint of MeOH, DCM and Hexane extracts: ^1^H NMR spectra of graviola extracts in CDCl_3_ at 400 MHz. (**A**) Spectrum of the *n*-hexane subfraction and expansion of its circled downfield segment at upper left of panel; (**B**) Spectrum of the DCM subfraction and expansion of its circled downfield segment rich in olefinic, aromatic, heteroaromatic, phenolic hydroxy and/or NH groups; and (**C**) MeOH extract spectrum in CD3OD and expansion of its circled downfield segment rich in aromatic and phenolic hydroxy groups.

The chemical composition of each of the three solvent extracts was investigated by ^1^H NMR spectroscopy to provide a preliminary overview of its constituents. The ^1^H NMR spectrum of the *n*-hexane extract (Figure 6A) indicated mono- and, to a lesser degree, poly-unsaturated fatty acids as the major organic constituents. There were also some downfield oxygenated signals consistent with mostly mono-oxygenated and possibly deoxygenated sterols. The ^1^H NMR data from the DCM extract (Figure 6B) suggest the presence of unsaturated fatty acids, in addition to extensive signals in the upfield and oxygenated regions, indicative of a complex mixture of different types of acetogenins, including mono and *bis*-tetrahydrofuran (THF), and mono-THF-tetrahydropyran (THP) acetogenins. There were also some aromatic and heteroaromatic protons at δ 6.90–8.50, in addition to far downfield δ 9.30–9.70 signals due to 6 or 7 NH signals, which are indicative of aporphine, protoberberine and/or isoquinoline alkaloids (the area from approximately δ 5–10 is enlarged at upper left of panel B). The ^1^H NMR spectrum of the MeOH extract (Figure 6C) indicates extensive aminosugar and glycoside contents displaying intense signals at the oxygenated proton region δ 3.00–5.30. There were also indications of flavonoids, polyphenols, and aromatic acids as evidenced by signals between δ 6.00–8.20 and several weak, about to be exchanged, phenolic hydroxyl and possibly NH groups at the most downfield δ values of 8.20–11.10.

**Figure 7:**
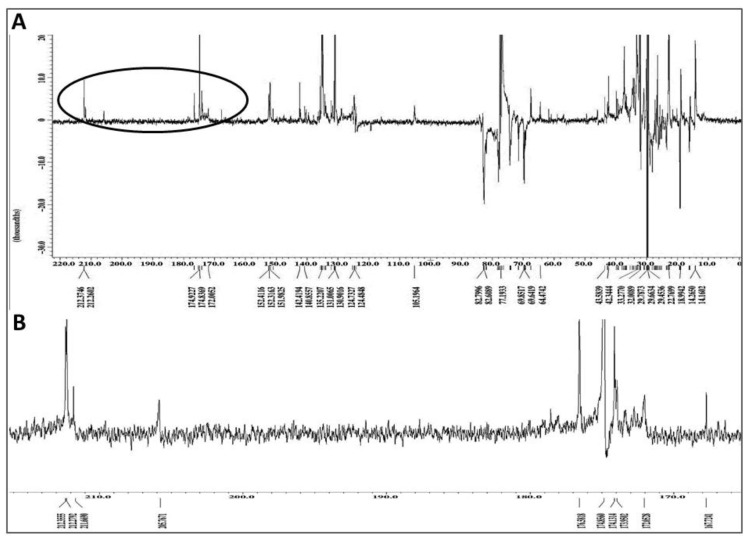
Chemical fingerprint of the dichloromethane (DCM) subfraction of GLSE. (**A**) Full PENDANT-^13^C NMR spectrum of the graviola DCM extract in CDCl_3_ showing methylene and quaternary carbons up and methine and methyl carbons down. The full spectrum shows four different signal clusters as explained in the text; and (**B**) Expansion of the circled portion of the spectrum from panel A containing lactone/ester carbonyl and ketone carbons clusters circled in PENDANT spectrum.

The PENDANT ^13^C NMR spectroscopy method is a J-modulation polarization transfer carbon sequence in which methylene and quaternary carbons are aligned up while the methyl and methine carbons are aligned down [36,37,38]. When applied to the graviola DCM extract (Figure 7A), four different signal clusters are apparent: an upfield aliphatic methyl and methylene carbons (δ 0–40), a cluster containing oxygenated methylene and methine carbons (δ 60–80), a group of methylenedioxy, olefinic and/or aromatic carbons (δ 105–152), and finally a group of lactone/ester carbonyl and ketone carbons (δ 165–212). The latter group is expanded in Figure 7B.

**Figure 8:**
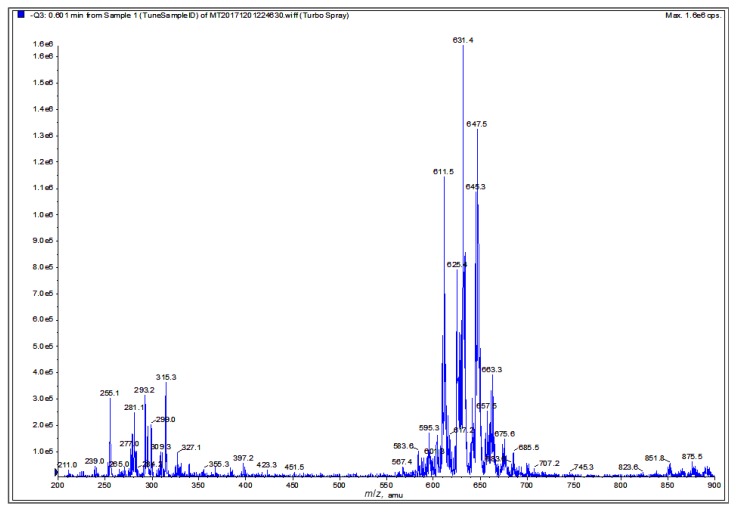
Chemical fingerprint of the graviola DCM extract. Electrospray ionization–mass spectrometry (ESI-MS) analysis of graviola DCM extract in negative ion mode. The cluster at *m*/*z* 567.4–685.5 is suggestive of acetogenin ion peaks while the cluster at *m*/*z* 239–327 is suggestive of potential alkaloid and smaller acetogenin ion peaks.

Finally, a negative ion mode electrospray ionization-mass spectrometry (ESI-MS) spectrum of the graviola DCM extract (Figure 8) shows an intense cluster at *m*/*z* 567.4–685.5, suggestive of acetogenin ion peaks, and a second cluster at *m*/*z* 239–327, which likely represents alkaloid and smaller acetogenin ion peaks (see also Discussion). These interpretations are based on the reported molecular weight data of graviola chemical class members [28,29,30,39].

## 3. Discussion

The steady increases in NMSC incidence that have been observed in the last few decades are likely due to a variety of predisposing risk factors including but not limited to inflammation, immune system deficits, nutrient deficiencies and genetic predisposition for developing NMSC [12,19]. Some success has been achieved by improvements in early diagnosis, though this has not translated into reducing NMSC morbidity and mortality. The main treatment modalities remain predominantly surgery and radiation, either of which can result in severe cosmetic issues in visibly exposed skin [25]. Therefore, NMSC patients and those at risk could benefit from chemotherapeutic and chemopreventive approaches with non-toxic, bioavailable natural agents.

Compelling scientific evidence indicates that regular consumption of fruits and vegetables is associated with decreased risk of developing chronic diseases including cancer [40,41,42,43,44,45]. The presence of a wide range of natural phytochemicals in plants, fruits and vegetables clearly confers multiple health benefits when consumed regularly. Bearing in mind that carcinogenesis is a multistep process, it would be unlikely that any individual agent could sufficiently tame this prevalent disease. However, the synergism of interactions between dietary nutraceuticals could significantly curb or prevent cancer by targeting multiple pathways. The non-toxic nature of these natural dietary compounds combined with the fact that they are already a regular part of the human diet provides built-in advantages over agents that are not usually consumed by the human population [26,30,39,45].

Given their complex composition, whole foods may provide benefits that exceed those of isolated single nutraceuticals, considering that certain fractions of entire extracts enriched in phytochemicals such as acetogenins, anthocyanins, carotenoids and polyphenols possess greater efficacy compared to their isolated individual ingredients [39,45]. Indeed, studies have shown that the complex interplay between the phytochemicals present in extracts from various fruits or plants has proven to possess greater anti-carcinogenic activities than any individual or purified ingredients [46], which is attributable to the concurrent targeting of multiple pathways resulting in superior chemopreventive effects [45,47,48,49].

The current study focuses on graviola (*Annona muricata*), a lowland tropical fruit-bearing tree of the family Annonaceae found in the rainforests of Africa, South America, and Southeast Asia [39]. In this study, we probed the molecular basis of the anti-cancer activity of the phytochemical-rich GLSE extract against two distinct types of NMSC cancer cells lines, the basal cell-derived UW-BCC1, and the squamous cell-derived A431, in vitro. Our data demonstrate that GLSE treatment of both NMSC cell lines results in cell growth arrest, inhibition of colony formation and wound healing, as well as alteration in molecules regulating the cell cycle (predominantly the G_1_ phase) and apoptosis (Figure 1, Figure 2 and Figure 3), with most of these effects being both dose-dependent and statistically significant. It is also noteworthy that most of these effects were evident at doses of GLSE that were far more effective at limiting growth in cancer cells vs. non-cancerous control NHEKn cells (see Figure 1B,C).

A major avenue of tumorigenesis is thought to involve dysregulated cellular proliferation leading to cellular expansion and accumulation of tissue mass. In eukaryotes, the cell cycle transition through different phases is coordinated by a family of protein kinase complexes, encompassing several cyclin dependent kinases (CDKs) and their activating partners, the cyclins [50,51]. The interaction of cyclins including cyclin D with CDKs 2, 4 or 6 leads to the phosphorylation and release of retinoblastoma (RB) from elongation factor 2 (E2F), resulting in cell cycle progression and cell growth [50,52,53]. Our data demonstrate an ability of GLSE to inhibit the expression of cyclins D and E and CDKs 2, and 4 alongside an induction of CDK inhibitors p21WAF1 and p27kip1 in GLSE treated cells. Upregulation of p21 and p27 would be expected to promote cell cycle withdrawal by blocking the activity of the cyclin/CDK complexes [54]. Additionally, both GLSE-treated NMSC cell lines displayed arrest in the G_0_/G_1_ phase of the cell cycle (Figure 2). We believe that these findings are significant for the reason that cell cycle regulation is an important target for prevention of cancers including non-melanoma skin cancers [39].

However, a dysregulated cell cycle is merely one part of cancer prevention and treatment [55]. Our current understanding of tumor biology also includes aberrant cell survival, and failure to induce apoptosis, both of which contribute to the transformed state. Indeed, several current chemotherapeutic approaches are designed to selectively trigger precancerous and tumor cell death while sparing non-cancerous cells [56]. Our data show that GLSE treatment results in the cleavage and inactivation of PARP, presumably as a consequence of activating the extrinsic and the intrinsic apoptotic pathways (Figure 3C). We also examined Bcl-2, which suppresses apoptosis and is highly expressed in most human tumors. Bcl-2 forms a heterodimer complex with Bax, neutralizing the pro-apoptotic effects of the latter [53]. Our results, which indicate that GLSE treatment mediates an increase in Bax expression and a corresponding down-regulation of Bcl-2, suggest that this may be a possible route through which GLSE induces apoptosis in non-melanoma skin cancer cells (Figure 3C). Our data are in accordance with previous reports showing that extracts of different graviola parts induce apoptosis in other cancer cell lines such as prostate [57], colon [58], breast [59] and leukemia cells [60], and reviewed in [39].

In adults, the hedgehog (Hh) pathway is mainly inactive under normal conditions, with the exception of its roles in tissue maintenance and repair [12,13]. The main components of this pathway, the Hh ligands Sonic, Indian, and Desert, bind to the receptor Patched (Ptch), and relieve the inhibition of the receptor protein Smoothened (Smo), leading to downstream signaling via the glioma-associated transcription factors, Gli 1 and Gli 2 [23]. Deregulation of the Hh pathway is very commonly associated with uncontrolled neoplastic growth; in fact, nearly 90% of BCC cases harbor loss-of-function mutations in at least one allele of Ptch and an additional 10% have gain-of-function mutations in Smo [23,61]. Therefore, inhibition of this pathway in NMSCs is a compelling therapeutic target. Two recently developed, FDA-approved, small molecule Hh pathway inhibitors (HPIs), namely vismodegib (GDC-0449; approved in 2012) and sonidegib (LDE225; approved in 2015) [62], act as Smo antagonists to block downstream transcriptional activation of the Hh pathway [62,63,64]. Although both of these HPIs display efficacy in eliminating disfiguring surgeries in patients with advanced BCC [65], several/serious adverse side-effects have been observed in BCC patients treated with these agents [66], including loss of taste buds [67], hair-loss, weight loss, and fatigue. The mechanisms behind these side effects are not yet understood, but these side effects frequently lead to a decrease in patients’ quality of life and even to discontinuation of treatment. Moreover, increased risk and occurrence of cSCC has been reported in patients after receiving vismodegib therapy for BCC [68]. Thus, more research to identify and develop novel, more efficient and safer strategies, especially with natural product supplements such as graviola that are already being consumed by the human population, is urgently needed for chemoprevention and chemotherapy of these cancers.

Here, we demonstrate that GLSE suppresses the activated hedgehog (Hh) pathway (Figure 4), via the inhibition of Smo, Gli 1 and Gli 2, with concurrent induction of SuFu in both UW-BCC1 and A431 cells. These actions lead to reductions in cell growth, clonogenicity and wound healing along with an induction of apoptosis, suggestive of a mechanism likely similar to that of the HPIs already in use. Furthermore, graviola extracts exert inhibitory effects on cancer cells with limited effects on normal cells; this finding, plus the low doses at which GLSE exerts its effects (particularly on Smo), suggests that GLSE components could possibly avoid the side effects of available HPIs.

Finally, after finding that GLSE possesses significant anti-proliferative, pro-apoptotic and anti-migratory activity in both UW-BCC1 and A431 cells, in vitro, we examined the potency of graviola subfractions extracted with different solvents. Qualitative analysis of *n*-hexane, dichloromethane and methanolic extracts revealed that each subfraction represents a complex mixture of chemical constituents with varying efficacy in preventing cell growth. However, the DCM fraction yielded by far the lowest IC_50_ values and hence the highest potency. These findings emphasize the therapeutic potential of GLSE, and it is tempting to attribute these effects, at least in part, to the high concentrations of acetogenins and alkaloids in the DCM subfraction. The exact chemical compositions of the fractions are yet to be defined and a full characterization is beyond the scope of the current study, which mainly employed ^1^H NMR and ESI-MS techniques to preliminarily characterize the major components in each subfraction (Figure 6, Figure 7 and Figure 8). ^1^H NMR data confirmed that the hexane extract contained the least polar and most lipophilic metabolites, namely sterols and fatty acids. Additionally, the DCM extract possesses ingredients of intermediate polarity, likely including multiple acetogenins and alkaloids [28,29,30], while the MeOH extract possesses the most polar components, likely consisting of flavonoids, acids, and aminosugars.

Since the DCM extract was the most biologically active fraction, it was further subjected to a PENDANT ^13^C NMR spectral analysis using a relaxation time of three seconds to offer more time for quaternary carbon relaxation to improve detectability and sensitivity, as well as ESI-MS analyses (Figure 7 and Figure 8, respectively). In addition to critical carbons consistent with either acetogenin or alkaloidal classes of compounds, the existence of seven lactone carbonyl carbons correlated with the number of expected major acetogenins. This finding further confirmed the presence of excessively oxygenated methine and methylene carbons at δ 60–80 in PENDANT spectrum (Figure 7), potentially suggesting the presence of mono-THF, mono-THF-mono-THP, and *bis*-THF acetogenins. The oxygenated quaternary aromatic carbons at δ 142–152 correlate with functionalized aporphine and protoberberine type alkaloids, which is consistent with the number of downfield NH protons at δ 9.30–9.70. The four-ketone carbons at δ 205–212 also suggest the presence of ketone-containing acetogenins.

The ESI-MS spectrum of the DCM extract (Figure 8) displays a cluster at *m*/*z* 567.4–685.5, implying the potential identity of acetogenins known to occur in graviola. For example, the molecular ion peak at *m*/*z* 611.5 could correspond to annopentocins A–C, muricatocins A–C, and/or muricapentocin [M-H]^+^, the molecular ion peak at *m*/*z* 595.3 may correlate with (+)-annonacin/annonacin A, gigantetrocins A/B, goniothalamicin, and/or javoricin [M-H]^+^, and the molecular ion peak at *m*/*z* 567.4 may represent muricins D and E [M-H]^+^ [28,29,30,31,39].

Another ESI-MS cluster at *m*/*z* 211–397.2 suggests the presence of known alkaloids in the DCM extract. For example, the molecular ion peak at *m*/*z* 265.3 could be attributed to anonaine, [M]^+^, the ion peak at *m*/*z* 281.4 may indicate the presence of nornuciferine, and the ion peak at *m*/*z* 285.3, [M]^+^, may represent coclaurine [M-H]^+^. Similarly, the molecular ion peak at *m*/*z* 299.0 could be tentatively ascribed to (*R*)-4´-*O*-methylcoclaurine, *N*-methylcoclaurine, and/or *N*-methylcoculaurine, [M]^+^, and the ion peak at *m*/*z* 309.3 could represent atherospermine, atherosperminine, and/or isolaureline, [M]^+^. Finally, the ion peak at *m*/*z* 327.4 could be coreximine [M]^+^ [31,39].

Our study provides evidence that a graviola leaf and stem extract (GLSE) and its different fractions can effectively inhibit NMSC (especially UW-BCC1) cell proliferation, induce apoptosis and also modulate the Hh pathway. Consistent with our findings, other laboratories have reported that GLSE can suppress pancreatic cancer cell activity [31], graviola pulp extract can inhibit prostate cancer cells [69], and that a graviola fruit extract can inhibit breast cancer cell growth [70]. Herein, we report that these effects are likely the result of targeting multiple pathways that regulate cell growth, survival and metastasis.

More rigorous fractionation and chemical characterization analyses are warranted to identify and establish the exact bioactive acetogenins and other ingredients and their relative amounts in various graviola dietary supplements, juices and foodstuffs, which are already widely consumed by humans with little or no toxicity. Once identified, the more potent active phytochemicals could then be used as lead compounds for anticancer drug development. Following chemical profiling, in vivo pharmacology studies will be necessary to ascertain the best combination of fractions and/or individual active ingredients/compounds for targeting deregulated molecular targets for cancer treatments.

Other strategies to be pursued in future studies include testing graviola components in comparison with or even in combination with FDA approved drugs like vismodegib and sonidegib. Indeed, we recently reported that vismodegib, one of only two FDA approved small molecule inhibitors for BCC treatment, inhibits colony formation by UW-BCC1 in a manner similar to what we observed with graviola extract [11].

## 4. Materials and Methods

### 4.1. Chemicals, Reagents and Antibodies

MTT dye (3-(4-dimethylthiazol-2-yl)-2,5-diphenyltetrazolium bromide, 98% TLC), Corning Transwell polyester membrane cell culture inserts, dimethyl sulfoxide (DMSO), and β-actin antibody were purchased from Sigma–Aldrich Chemical Company (St. Louis, MO, USA). The antibodies for Smoothened (Smo), Suppressor of fused homolog (SuFu), Sonic hedgehog (Shh) and Glyceraldehyde 3-phosphate dehydrogenase (GAPDH) were purchased from Santa Cruz Biotechnology, Inc. (Santa Cruz Co., Santa Cruz, CA, USA). The antibodies for Gli 1 and Gli 2 were purchased from Abcam (Cambridge, MA, USA). Antibodies against cyclin-dependent kinase 2 (cdk2), cdk4, cyclin D1, cyclin E1, p21WAF1, p27kip1, Poly (ADP-ribose) polymerase (PARP), PARP (9542S), Bcl-2, Bax, phospho-Akt (Ser 473), Vinculin, Caspase-3, Cleaved Caspase-3 (Asp175) (5A1E), Cleaved Caspase 8, and horseradish peroxidase-conjugated (HRP) anti-mouse and anti-rabbit secondary antibodies were all obtained from Cell Signaling Technology (Beverly, MA, USA). Mini-protean precast Tris-Glycine gels were from BioRad (Hercules, CA, USA). An enhanced chemiluminescence (ECL) detection system was from GE healthcare (Buckinghamshire, UK). A 2% (*w*/*v*) Aqueous Solution of Gentian Violet was from Ricca Chemical Company (Arlington, TX, USA). Invitrogen Novex precast Tris-Glycine gels, and Dulbecco’s modified Eagle’s medium (DMEM) were from Corning. Eagle’s minimum essential medium (EMEM) with nonessential amino acids and L-glutamine, but without calcium, a Human Keratinocyte Growth Supplement Kit (HKGS Kit), Trypsin neutralizer solution, Pierce BCA™ protein assay kits, Pierce SuperSignal^®^ West Pico chemiluminescent substrate kits and Promega™ Caspase-Glo™ 3/7 Assay Kit were all procured from Thermo Fisher Scientific (Rockford, IL, USA). Fetal bovine serum (FBS) was from Hyclone (Pittsburgh, PA, USA), EpiLife^®^ Medium with 60 µM calcium was from Life Technologies; penicillin-streptomycin-amphotericin B (PSA) was from Mediatech Inc. (Herndon, VA, USA); Annexin V FITC Fluorescence Microscopy Kit (Cat# 550911) and the APO-DIRECT KIT (Cat#556381) were from BD Biosciences (San Jose, CA, USA); Trypsin/EDTA Solution (TE) was from Life Technologies; the 8.0 µm pore size trans-well membrane inserts were from BD Bioscience (Cat# 353097). Organic solvents including dichloromethane, acetic acid, ethyl acetate, ethanol (EtOH), methanol (MeOH) were purchased from VWR (Suwanee, GA, USA), dried by standard procedures, packaged under nitrogen in Sure/Seal bottles and stored over 4 Å molecular sieves. All other chemicals were purchased from Sigma-Aldrich (St. Louis, MO, USA), unless otherwise stated.

### 4.2. Preparation of Graviola Leaf and Stem Extract (GLSE) and Successive Extractions

Graviola dietary supplement capsules were purchased from Rainforest NP Inc. (Rainforest Pharmacy, Miami, FL, USA). Each capsule consisted of 500 mg of 100% pure, finely milled graviola leaf and stem powder with no binders or fillers. For initial whole extract studies, the capsule contents were suspended in DMSO (500 mg/mL) and incubated for 10 min at 30 °C under constant swirling and shaking. The suspension was carefully vortexed, centrifuged and the supernatant was filtered to remove any remaining particles. A primary graviola leaf-stem extract (GLSE) stock solution was prepared at 100 mg/mL and stored at −80 °C. A secondary stock solution (10 mg/mL) was prepared in media, and further dilutions were made freshly from this secondary stock solution into the respective growth media prior to treatment of specified cell lines.

To prepare successive solvent extracted subfractions, 20 graviola aerial part capsules (500 mg each, 10 g total contents) were unpacked, macerated for 60 min, then extracted with 100 ml of either: (1) *n*-hexane, (2) dichloromethane (DCM), or (3) methanol (MeOH). Each solvent extract was filtered, evaporated under vacuum, and each subfraction was freeze-dried and stored frozen under liquid nitrogen until used. Freeze-dried powders were then dissolved in DMSO and stored at −20 °C for further use.

### 4.3. Cell Lines, Culture and Treatment Conditions

Two human NMSC cell lines were employed in this study; our previously established and characterized superficial basal cell carcinoma cell line, UW-BCC1 [13], and a cutaneous squamous epidermoid carcinoma cell line, A431. As controls for normal non-cancerous epithelial cells, neonatal primary normal human epidermal keratinocytes (NHEK) [67] were used. UW-BCC1 cell were maintained in EMEM medium with 5% FBS, further supplemented with HKGS Kit and 1% PSA. A431 cells were purchased from the American Type Culture Collection (ATCC, Manassas, VA, USA), and were cultured in DMEM supplemented with 5% heat-inactivated FBS and 1% PSA. NHEKs were cultured in keratinocyte Epi-Life serum-free medium supplemented with HKGS Kit as described earlier [68]. All cell batches were expanded, tested for mycoplasma contamination and frozen down for future use (every 2 months from a frozen vial) to ensure that cells used in experiments were at less than 20 passages from procurement. Cells were incubated in a 95% humidified atmosphere with 5% CO_2_ at 37 °C. Growth media were replenished on alternate days until reaching desired confluence (60–80%) prior to experimental treatments. The cells were incubated with various concentrations of GLSE (1–160 µg/mL) or different solvent fractions (1–100 µg/mL). Untreated growth media containing vehicle DMSO (0.01%) were utilized as negative controls for all assays unless otherwise indicated.

### 4.4. Cell Growth/Proliferation and Viability Assays

For the MTT assay, UW-BCC1, A431 and NHEK cells were seeded in 96-well microtiter plates at a density of 5000–10,000 cells per well in 200 μL of culture media. After an overnight incubation to allow for cell adherence and proliferation, growth media were replenished with media containing various concentrations of GLSE (0–160 μg/mL) or various concentrations of each of the different solvent extracted fractions (0–100 μg/mL) for 24 and/or 48 h. After incubation, MTT (stock solution in phosphate-buffered saline (1× PBS) [5 mg/mL], was reconstituted in growth media to 0.5 mg/mL, and 100 μL was added to each well and incubated for 3 h. Plates were then centrifuged at 180× *g* for 5 min at 4 °C and the supernatant was discarded. The purple tetrazolium crystals were dissolved in 100 μL of DMSO and incubated in the dark under slow shaking. The absorbance was recorded at 570 nm on a Synergy™ Biotek multi-well microplate plate reader (BioTek, Winooski, VT, USA).

During our analysis of cells using MTT, we discovered that up to 35% of the UW-BCC1 cells became detached after manipulations and washes. Because MTT assay requires media changes, we resorted to a different method for assessing UW-BCC1 viability, namely the Cell Counting Kit-8 (CCK-8; Dojindo Molecular Technologies, Inc. Washington, DC, USA). After semi-adherent UWB-BCC1 cells reached 80% confluence, the CCK-8 kit was utilized following the manufacturers recommendation. This kit is based on WST-8 (2-(2-methoxy-4-nitrophenyl)-3-(4-nitrophenyl)-5-(2,4-disulfophenyl)-2*H*-tetrazolium, monosodium salt), which produces a water-soluble formazan dye upon bio-reduction in the presence of an electron carrier, 1-methoxy PMS. Since the CCK-8 solution is very stable and it has little cytotoxicity, a long incubation with the cells (24 to 48 h) was possible in order to determine number of viable cells. Each experiment was repeated at least 7 times in quadruplicate with similar results. The effect of graviola extracts on growth inhibition was calculated as % cell viability, with viability of DMSO-treated cells (untreated controls) set at 100%. IC_50_ values (concentrations which inhibited 50% of cellular growth) were determined by plotting an absorbance versus concentration curve.

In the trypan blue dye exclusion assay, the effect of GLSE on cell growth inhibition/viability was also assessed as percent viable cell number. Both UW-BCC1 and A431 cells were seeded in triplicate at 2 × 10^5^ cells per 100 mm petri-dish and incubated for 24 h to allow for adherence. Cells were treated in the presence or absence of GLSE (0–160 µg/mL) for 48 h prior to harvest by trypsinization, and cell proliferation was evaluated as follows: total live cell and dead cell numbers were determined using a TC20 automated cell counter (BioRad) after trypan blue staining. Cells were evaluated for percent viable cell number harvested at 48 h in relation to the percentage obtained at the beginning of treatment (0 h), and DMSO (0.01–0.05%) vehicle-treated cells were normalized as 100% viable. The experiment was repeated three times, each in triplicate.

### 4.5. Scratch Wound Healing Assay (SWHA)

UW-BCC1 and A431 cells were each seeded at a density of 5 × 10^4^ cells in triplicate in 12-well tissue culture plates in their respective growth media. After overnight incubation, a linear artificial wound was created on 100% confluent cell monolayers by scraping using sterile Gilson pipette tips. Media containing GLSE (0, 15, 30, 60, and 90 μg/mL, depending on cell line) were then added. The motility of cells across the wound and/or closure of the artificially inflicted wound were monitored in each treatment group using a Zeiss light microscope. Light microscopic images (20× and 40×) were captured immediately after adding GLSE (0 h), and after 30 h of treatment. The distances between the edges of defect (wound) were measured, and average values were determined as an indicator of the progress of wound healing over the 30 h period according to previously described protocol [69,70,71].

### 4.6. Colony Formation Assay

Colony formation (clonogenic) assays were performed on pre-confluent UW-BCC1 and A431 cells, which were treated with or without varying concentrations of GLSE (0–160 μg/mL) for 48 h. Following treatment, the cells were trypsinized and re-seeded in triplicate at appropriate dilutions (~3000 cells/100 mm tissue culture petri-dish) in 10 mL of drug-free growth media. Cultures were allowed to reinitiate colony in regular growth medium with media change after three-days and subsequently every alternate day as colony densities increased. After 12–16 days of growth, at a time when cell colonies from control culture attained maximum confluence, the cells were washed twice with ice cold 1xPBS followed by combined fixation and gentian-violet staining. Briefly, a solution consisting of 4% paraformaldehyde and 0.5% gentian-violet in methanol was added to the cells and allowed to incubate at RT for 1 h. Gentian violet solution was discarded and cells were washed in tap water, then deionized water, air-dried and photographed using a digital camera. Colonies were counted under the microscope. Photos were enhanced using Adobe Photoshop for brightness and contrast, and sharpened for uniformity of appearance. All experiments were repeated three times.

### 4.7. Trans-Well Migration/Motility Assay

The effect of GLSE on the trans-migration of UW-BCC1 and A431 cells was analyzed using a trans-well migration assay using 8-µm trans-well inserts (BD Bioscience). UW-BCC1 and A431 cells (5 × 10^5^) were suspended in culture media containing GLSE (0–160 μg/mL) and reduced FBS (1%), and were seeded onto 8 μm pore size polyethylene terephthalate (PET) membranes. Appropriate culture media were then supplemented with 10% FBS and added to the bottom of each well and incubated for 48 h. After incubation, the cells remaining on the upper membrane were removed by cotton swabs, whereas the cells that migrated to the bottom of the PET membrane were fixed and stained with 0.5% gentian-violet as above, air-dried and counted. The number of cells that passed through the membranes was quantified by counting 20 random fields under light microscopy at 100× magnification. Each condition was tested in three separate wells from three independent experiments. For quantification, gentian violet was dissolved in 50% acetic acid and absorbance at 540 nm was measured.

### 4.8. Protein Extract Preparation and Immunoblot Analysis

Growth media from UW-BCC1 and A431 cells, treated with or without GLSE (0–160 μg/mL) for 48 h, were aspirated, and the cells were washed with ice-chilled PBS (pH 7.4). Washed cells were incubated in ice-cold lysis buffer (50 nmol/L Tris-HCl, 150 mmol/L NaCl, 1 mmol/L EGTA, 1 mmol/L EDTA, 20 mmol/L NaF, 100 mmol/L Na_3_VO_4_, 0.5% Nonidet P-40, 1% Triton X-100, 1 mmol/L PMSF, pH 7.4) with freshly added Protease Inhibitor Cocktail Set III (Calbiochem, La Jolla, CA, USA) on ice for 15 min. The cells were then scraped and lysates were collected into a microfuge tube and passed through 22.5-gauge syringe needles to break up the cell aggregates as previously described [71,72,73]. The lysate was cleared by centrifugation at 14,000× *g* for 30 min at 4 °C, and the supernatant (whole cell lysate) was either immediately used or aliquoted and stored at −80 °C for further analysis. Protein concentrations for each lysate were determined using the BCA protein assay kit according to the manufacturer’s protocol.

For Western blotting, protein lysates (approximately 20–30 μg of protein) were denatured in 2× Laemmli sample buffer and subjected to electrophoresis on 8–12% sodium dodecyl sulfate–polyacrylamide gels (SDS-PAGE) or Tris–glycine gels as previously described [71,72]. The separated proteins were transferred onto nitrocellulose membranes followed by blocking with 5% non-fat milk powder (*w*/*v*) in Tris-buffered saline (10 mM Tris-HCl, pH 7.5, 100 mM NaCl, 0.1% Tween 20) for 45 min at room temperature as earlier described [71,72,73]. Membranes were probed for proteins of interest using specific primary antibodies followed by the appropriate peroxidase-conjugated secondary antibody. The blots were exposed to enhanced chemiluminescence (ECL) and subjected to autoradiography using a BioRad imaging system. To ensure equal protein loading, membranes were stripped and re-probed with appropriate loading controls. Densitometric analyses of the visualized protein bands were performed using the BioRad digitized scientific software program Quantity One. Bands were scanned and processed with Adobe Photoshop CS 6.0 (Adobe Systems, San Jose, CA, USA). Multiple exposures were performed to ensure a linear range of band densities. Three independent experiments were performed for each analysis and protein expression levels were analyzed in triplicate with comparable results. Final data were analyzed by one way ANOVA.

### 4.9. Detection of Caspase-3 and -7 Activity

Apoptotic activity of GLSE-treated UW-BCC-1 and A431 cells was detected using the Caspase-Glo 3/7 activity apoptosis assay kit (Promega, Madison, WI, USA), according to the manufacturer’s instructions. In brief, cells were treated with media containing GLSE (0–90 μg/mL) and incubated overnight. Plates were then removed from the incubator and allowed to equilibrate to room temperature for 30 min, after which 100 μL of Caspase-Glo reagent mix was added to each well. The wells were gently mixed on a plate shaker at 300–500 rpm for 30 s and then incubated at room temperature for 3 h. The luminescence of each sample was measured in a Synergy™ Biotek multi-well microplate plate reader (BioTek, Winooski, VT, USA) using a 1 min lag time and 0.5 s/well-read time parameters. The experiments were performed in triplicate and repeated twice.

### 4.10. Cell Cycle and Apoptosis Assessment by Flow Cytometry/Immunofluorescence Microscopy

Induction of apoptosis by GLSE and GLS extracted subfractions in UW-BCC1 and A431 cells was analyzed by flow cytometry using the Apo-Direct kit and via fluorescent microscopy of Annexin V/PI staining. Briefly, UW-BCC1 and A431 cells were seeded, attached overnight, then incubated with or without GLSE (0–160 µg/mL) or DCM extract (0–10 µg/mL) for 24 h in their respective complete media prior to harvest and analysis.

For flow cytometry, cells were trypsinized, washed with PBS, and fixed with ice cold 1% paraformaldehyde in 1× PBS for 1 h, washed twice with cold 1× PBS and centrifuged at 1000 rpm for 5 min. The washed cell pellet was re-suspended in ice cold 70% ethanol for 1 h at −20 °C or stored overnight. Following resuspension and three additional cold PBS washes and centrifugation as above to remove ethanol, the cells were stained with FITC-labeled antiBrdUTP and propidium iodide using the Apo-Direct Kit (BD Biosciences) as per the manufacturer’s protocol. Cells were analyzed on a FACScan cytometer (Becton Dickinson, NJ, USA). A total of 10,000 gated single events were recorded each time, and cell-cycle distribution was analyzed using Flow CellQuest software (BD Biosciences) to determine the percentages of cells in G_0_/G_1_, G_2_/M, and S phases as well as those undergoing apoptosis. The experiments were repeated at least three times for each variable.

### 4.11. Apoptosis Assessment by Immunocytochemistry/Immunofluorescence Microscopy

For apoptosis analyses using the Annexin-V FITC Fluorescence Microscopy staining kit, apoptotic cells (Annexin V; green fluorescence), and necrotic cells (PI; red fluorescence) were analyzed as described below. UW-BCC1 and A431 cells (20,000 cells/chamber) were grown to about 60% confluence in 8-chamber slides and then treated with DCM extract as described earlier [71,72] for 48 h. After ice-cold methanol fixation, cells were blocked with 5% goat serum in PBS, and incubated with Annexin V-FITC (1:100 dilution) and propidium iodide (1:100 dilution), following the manufacturer’s protocol. This staining uses a dual-staining protocol in which the cells show green fluorescence of Annexin-V (apoptotic cells) and red fluorescence of propidium iodide (necrotic cells or late apoptotic cells). Fluorescence images (200× magnification) were captured using the EVOS FL cell imaging system with a color CCD camera (Life Technologies, Grand Island, NY, USA).

### 4.12. Nuclear Magnetic Resonance (NMR) Spectroscopic Analysis of Extracts

^1^H and ^13^C/PENDANT FT NMR spectra were recorded at 400 and 100 MHz, respectively, on a JEOL Eclipse ECS-400 NMR spectrometer (JEOL Inc., Peabody, MA, USA) equipped with a 5 mm FG/TH autotune probe in CD_3_Cl, except for the MeOH extract spectrum, which was acquired in CD_3_OD, using the residual solvent peak as internal reference [36,37,38,39]. A relaxation delay of three seconds was used in the PENDANT experiment instead of the default two seconds.

### 4.13. Mass Spectrometric (MS) Analysis of Graviola DCM Extract

ESI-MS experiments were conducted using an Applied Biosystems–MDS SCIEX API 3200^TM^ triple quadrupole LC/MS/MS system (Applied Biosystems, Foster City, CA, USA) equipped with a Turbo V^TM^ IonSpray^TM^ source using MS with an electrospray ionization (ESI) interface operated in negative ion mode using Analyst version 1.4.1 software (MDS Sciex, Toronto, Canada) [36,37,38]. For MS, about 1 mg of dry DCM extract was dissolved in 1 mL of HPLC-grade methanol, filtered and used for analysis.

### 4.14. Statistical Analysis

All statistical analyses were performed with GraphPad Prism™ version 6.1 (GraphPad Software Inc., San Diego, CA, USA). Statistical significance was determined using one-way ANOVA followed by Tukey’s test for multiple comparisons. Data are presented as the mean ± standard deviation (SD) with significance set at *p* values ≤0.05.

## 5. Conclusions

To our knowledge, the present study represents the first testing of graviola extracts with human non-melanoma skin cancer cells. Herein, we demonstrate that graviola leaf/stem extract (GLSE) can inhibit cell proliferation, motility and clonogenicity, induce apoptosis as well as suppress activated hedgehog pathway components in both UW-BCC1 and A431 cell lines. Furthermore, initial fractionations and chemical analyses suggest that the most potent activity of the powder is concentrated in acetogenin and/or alkaloid-rich dichloromethane (DCM) fractions. Further characterization is underway in our laboratories with the goal of identifying the most efficacious active constituents of the DCM subfraction. We plan to follow these studies with preclinical evaluation of GLSE, its subfractions and purified active compounds in murine xenograft models of BCC [11] and SCC with the goal of ultimately establishing graviola components as lead compounds or even as treatments in their own right for clinical trials. Testing these ingredients individually or in combination may lead to the development of novel agents for clinical management of these common forms of human skin cancers.

## Supplementary Materials

The following supplementary figures and legends are available online at http://www.mdpi.com/1422-0067/19/6/1791/s1.

## Abbreviations

NMSC	Non-Melanoma Skin Cancer
BCC	Basal cell Carcinoma
CDK	Cyclin Dependent Kinase
PARP	Poly ADP Ribose Polymerase
SHH	Sonic Hedgehog
Smo	Smoothened
Gli 1 & 2	Glioma-Associated Oncogene Homolog 1 & 2
SCC	Squamous Cell Carcinoma
DCM	Dichloromethane
MeOH	Methanol
PBS	Phosphate Buffered Saline
GLSE	Graviola Leaf and Stem Extract
NHEK	Normal Human Epidermal Keratinocytes
SuFu	Suppressor of Fused
