# Supplementary material for: Graviola (Annona muricata) Exerts Anti-Proliferative, Anti-Clonogenic and Pro-Apoptotic Effects in Human Non-Melanoma Skin Cancer UW-BCC1 and A431 Cells In Vitro: Involvement of Hedgehog Signaling
Source: Int J Mol Sci. 2018 Jun 16;19(6):1791. doi: 10.3390/ijms19061791 (PMC6032424; doi:10.3390/ijms19061791)
Supplement: Supplementary file 1 [file ijms-19-01791-s001.pdf]

Supplementary Figures and Figure Legends:

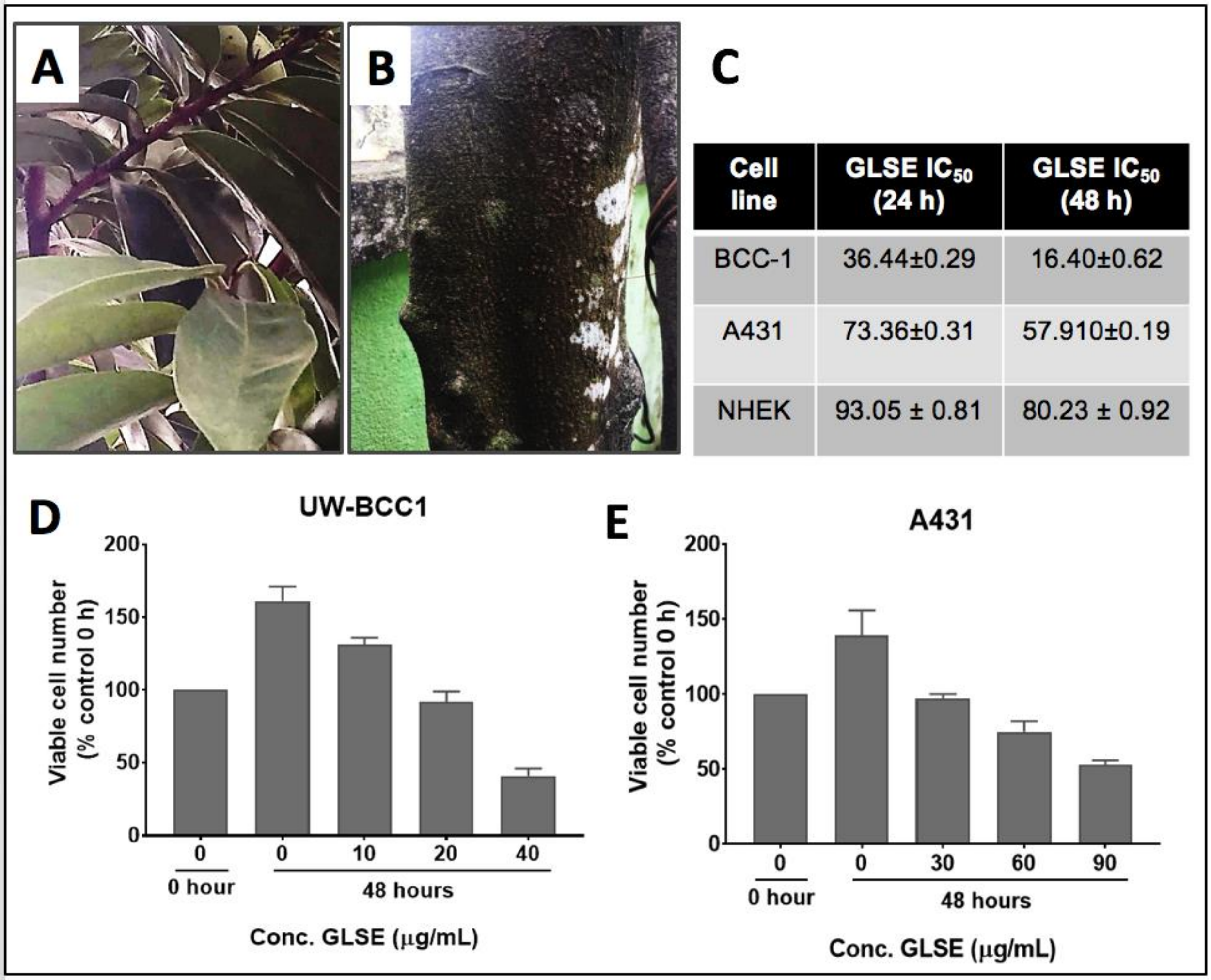

Cell line	GLSE IC_{50} (24 h)	GLSE IC_{50} (48 h)
BCC-1	36.44±0.29	16.40±0.62
A431	73.36±0.31	57.910±0.19
NHEK	93.05 ± 0.81	80.23 ± 0.92

Figure S1. ***Characterization of graviola (Annona Muricata) as a potential NMSC therapeutic.*** The aerial parts of graviola including (A) leaf and stem and (B) bark. Commercially available leaf and stem powder extracts of graviola were used throughout the experiments as described in the Methods section of the manuscript. (C, D and E) NMSC cells were treated with GLSE for 24 and 48h, and cell viability was determined by trypan blue dye exclusion assays (corresponding NMSC growth curves are shown in Figs. 1B and 1C in the regular text). (C) Table showing the IC_{50} values for GLSE with UW-BCC1, A431 and NHEK cells at 24 and 48h. Values shown are means ± SD of experiments performed three times with eight replications of each treatment. A trypan blue exclusion assay was used to determine the number of viable cells after 48 hours. The proportions of viable (D) UW-BCC1 and (E) A431 cells at 48h of incubation with the indicated doses of GLSE (μg/ml) in relation to the number at time zero (0 h), at which time the control was considered to be 100% viable. Mean ± SD of experiments performed in triplicate is shown, with P values represented by asterisks: *p <0.05 and **p <0.01 and ***, p < 0.001.

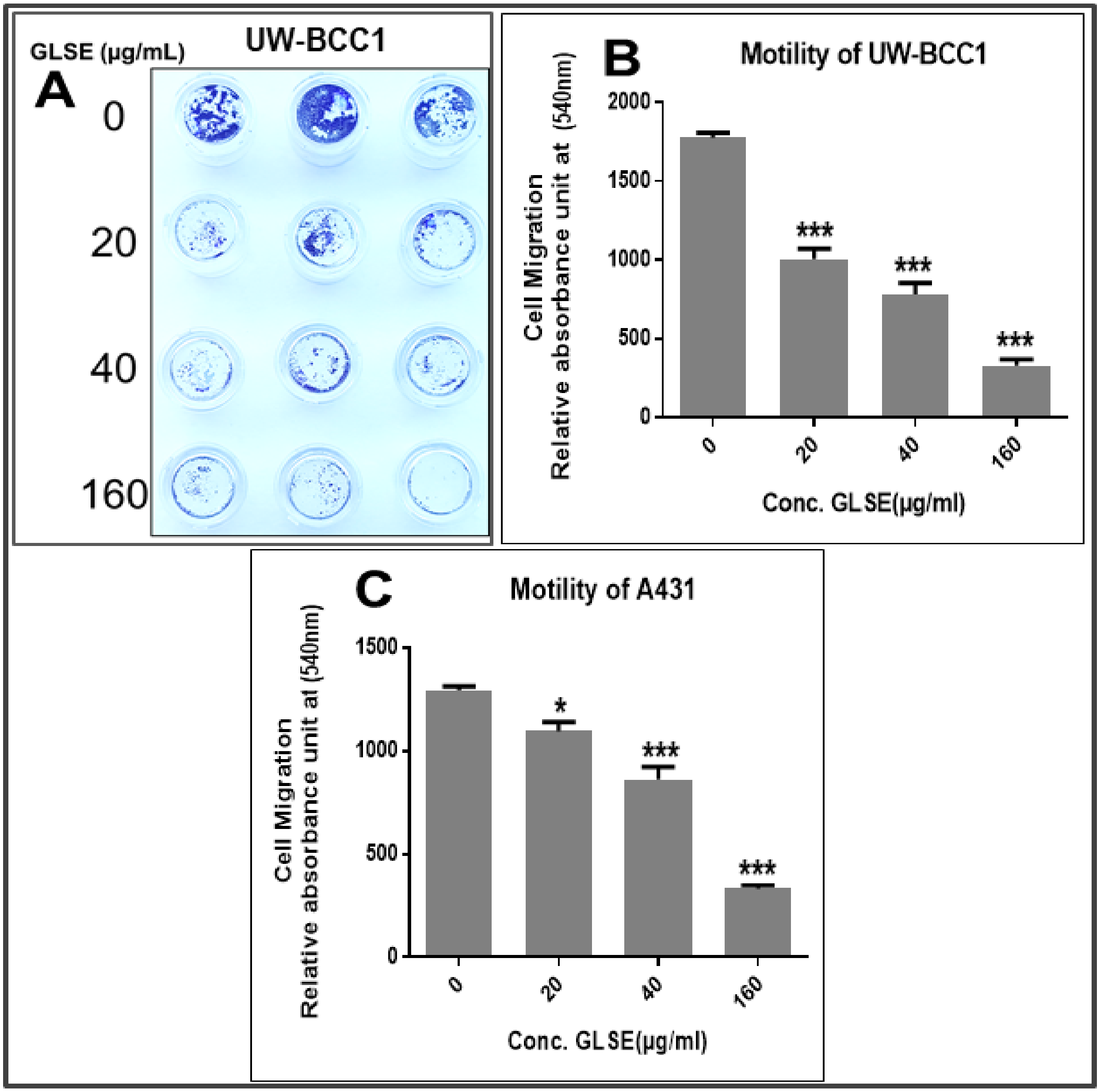

Figure S2. ***GLSE decreases the migration of UW-BCC1 and A431 cells.*** GLSE treatment induced a significant dose-dependent inhibition of transwell migration and motility of both UW-BCC1 and A431 cells. (A) Representative micrographs of transwells showing dose-dependent decreases in UW-BCC1 cell migration. Gentian violet absorbance of stained (B) UW-BCC1 and (C) A431 cells that migrated through the transwells were quantified and averaged. Error bars indicate SD of mean of three independent experiments performed in triplicate, *$p<0.05$ and **$p<0.01$ and ***, $p < 0.001$ compared to control cells receiving no GLSE treatment.

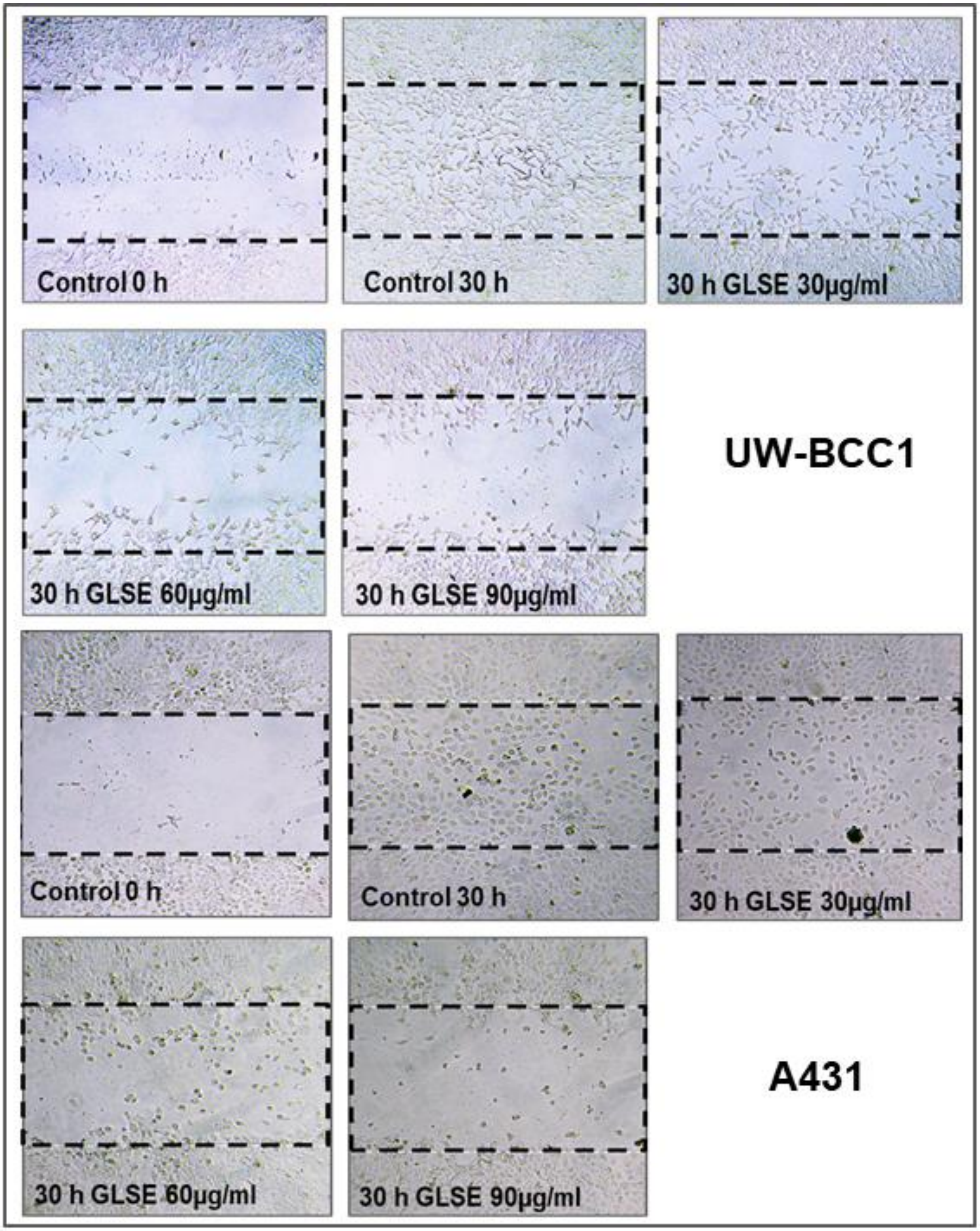

Figure S3. ***GLSE treatment inhibits cell invasion and scratch wound healing in UW-BCC1 and A431 cells.*** Images were taken at 0 hour and 24 hours after wound lines were created and compared to time 0 h. Treatment with GLSE for 24 h dose-dependently inhibits wound-healing (migration of cells to close artificial wound) of UW-BCC1 cells (top five panels) or A431 cells (bottom five panels).

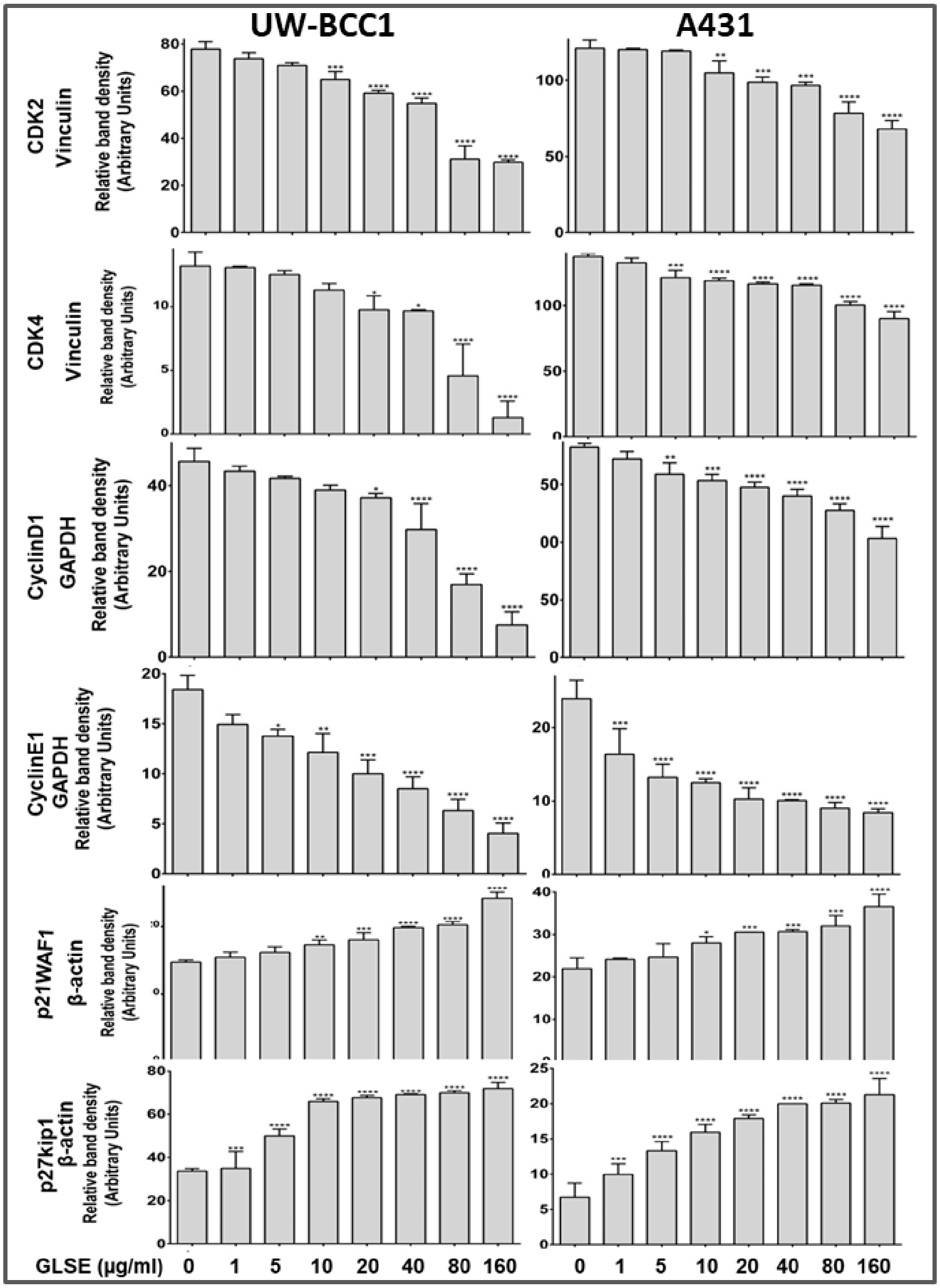

Figure S4. GLSE* significantly *induces G_0/G_1 phase cell cycle arrest* of *UW-BCC1 and A431 cells. Quantification of effects of GLSE treatment on cell cycle regulatory proteins. Whole cell lysates of UW-BCC1 and A431 cells with/without GLSE (0-160 μg/ml: 24h) were subjected to SDS-polyacrylamide gel electrophoresis. Blots were analyzed by immunoblotting with antibodies for CDK2, CDK4, Cyclin D1, Cyclin E1, p21/WAF1 and p27/kip1 and equal loading was confirmed by re-probing with GAPDH, β-Actin or vinculin as loading controls. The histograms shown are Means ± STDEV of three independent experiments with similar results. *p<0.05 and **p<0.01 and ***, p < 0.001.

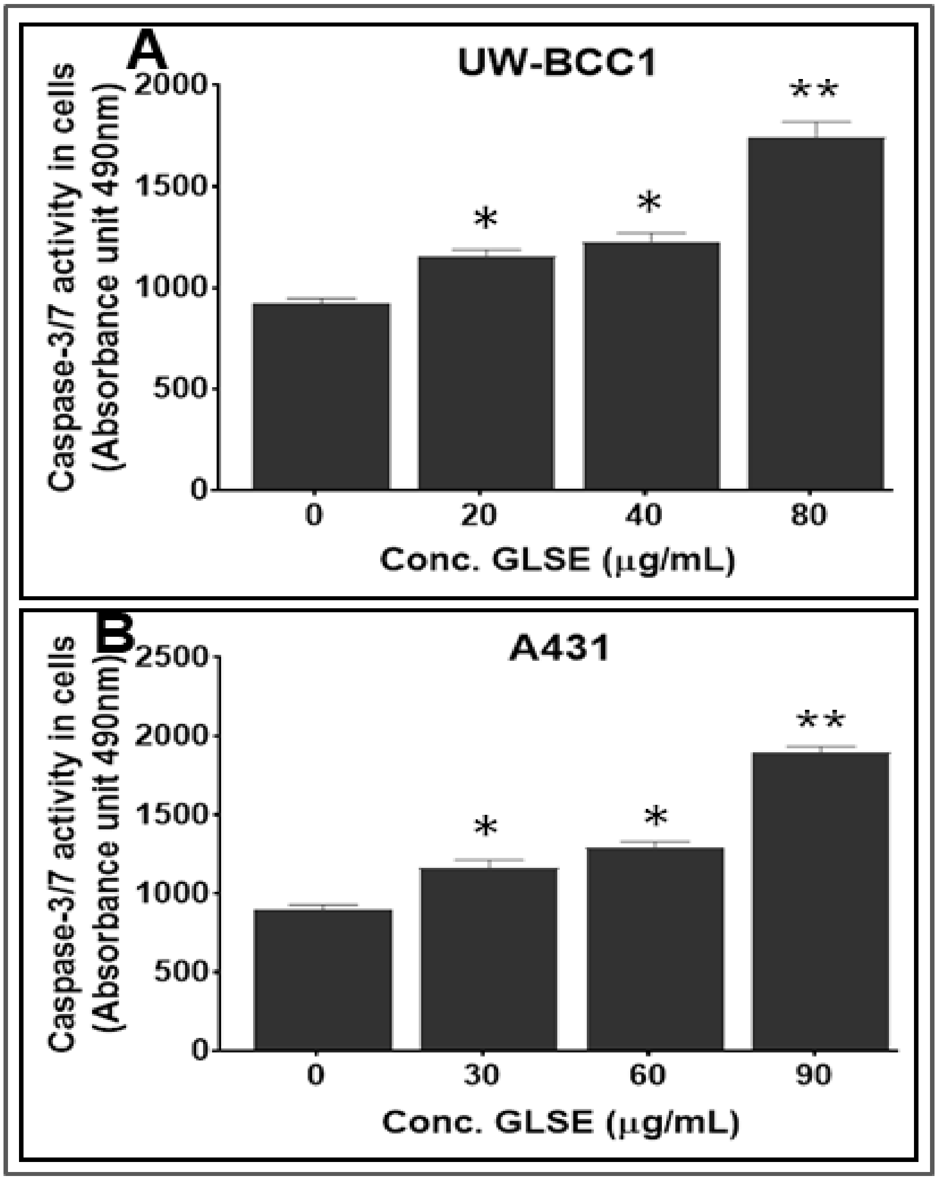

Figure S5. GLSE **significantly** ***increases caspase -3/-7 activity*** **of** ***UW-BCC1 and A431 cells.*** To further confirm the activation of the apoptotic pathway in GLSE treated UW-BCC1 and A431 cells, we first examined activation of caspase-3 and -7 activities in both (A) UW-BCC1 and (B) A431 cells after 24 h of treatment with different doses of GLSE. Error bars indicate SD of mean of three independent experiments performed in triplicate. *$p<0.05$ and **$p<0.01$ and ***, $p < 0.001$ compared to activity in untreated cells.

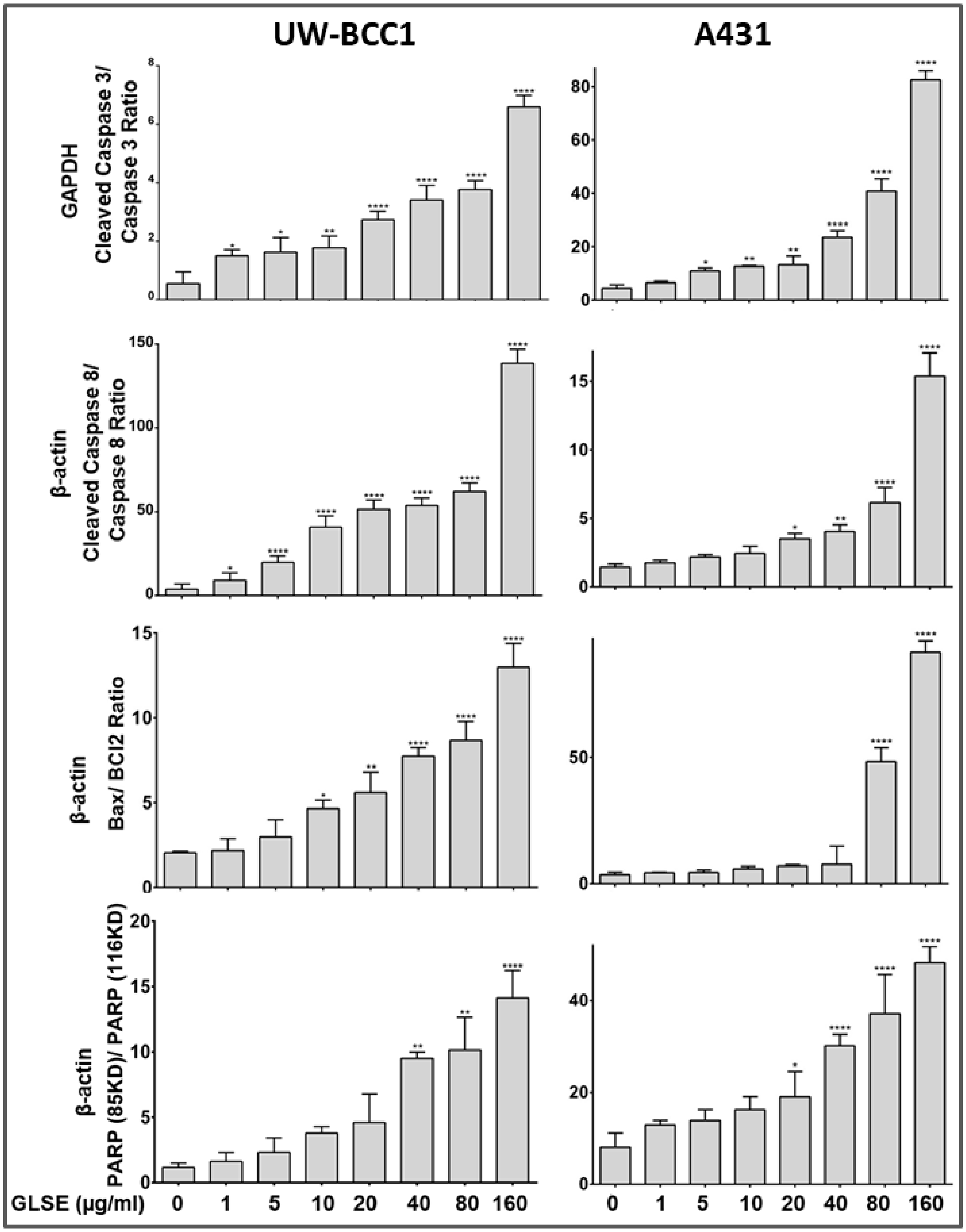

Figure S6. GLSE* significantly *modulates markers of apoptosis in UW-BCC1 and A431 cells. To investigate the mitochondria-mediated apoptotic pathway in GLSE treated UW-BCC1 and A431 cells, we examined the expressions of markers of apoptosis including caspase-3, -8, Bax, Bcl2, and PARP expression in both UW-BCC1 and A431 cells after 24 h of treatment with different doses of GLSE. The bar graphs represent normalization with house-keeping markers or the cleaved and preformed ratios. Error bars indicate SD of mean of three independent experiments performed in triplicate, *$p<0.05$ and **$p<0.01$ and ***, $p < 0.001$ compared to activity in untreated cells.

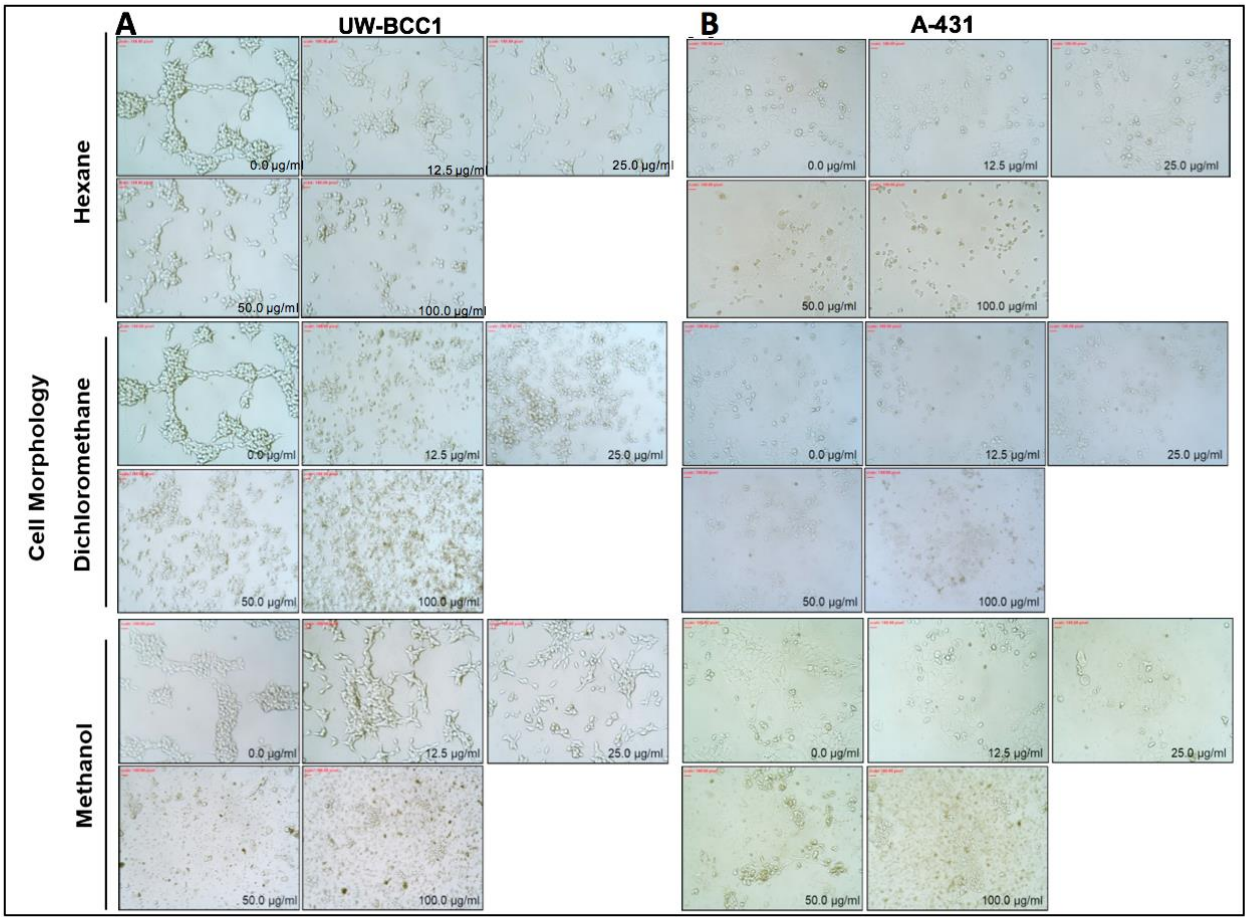

Figure S7. Solvent extracted subfractions of graviola* dose-dependently induce morphological changes in *UW-BCC1 and A431 cells. Photomicrographs showing morphological changes induced in (A) UW-BCC1 and (B) A431 cells upon treatment with different concentrations of graviola fractions extracted with hexane, dichloromethane or methanol, as indicated in the figure.

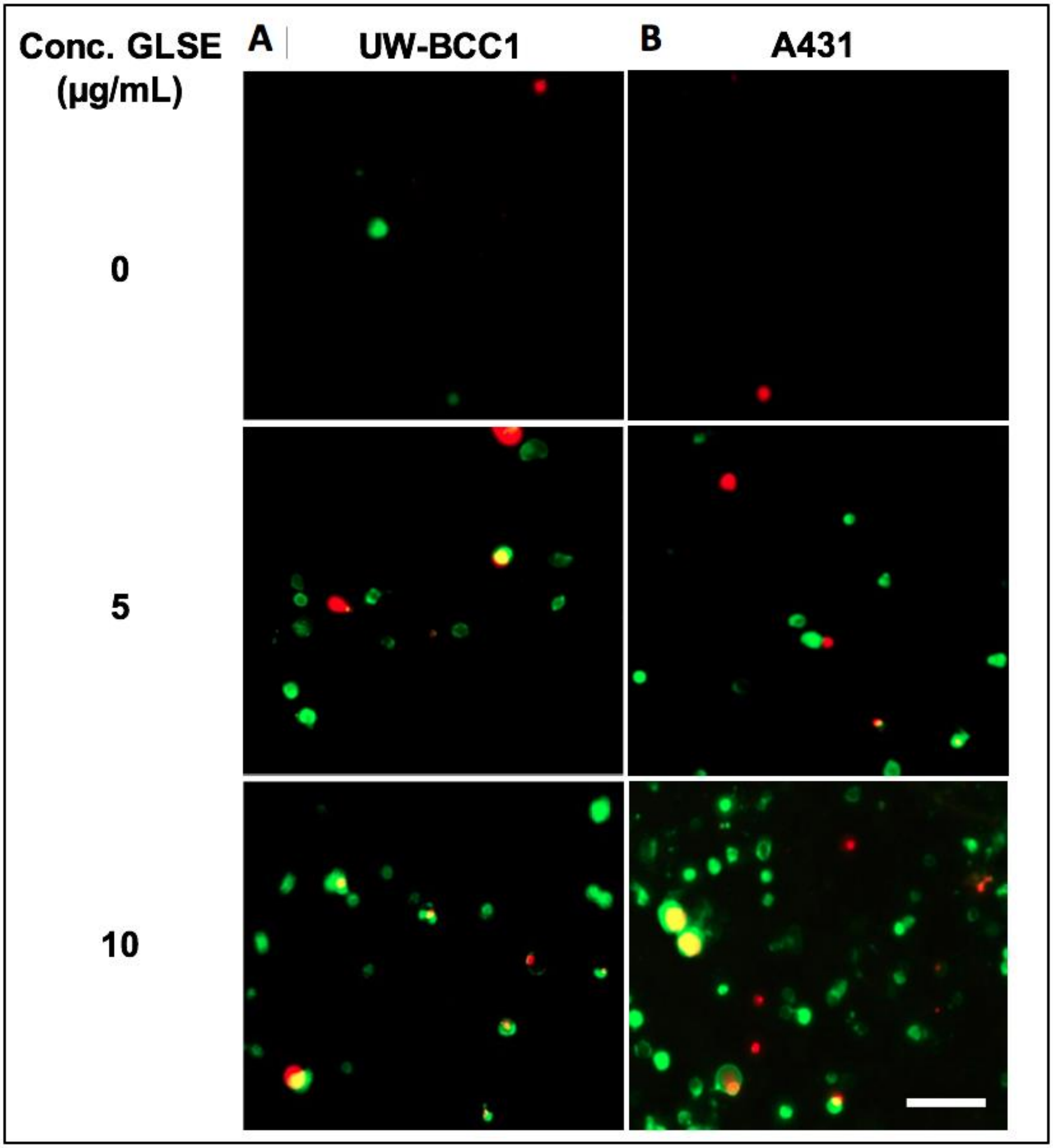

Figure S8. Effect of treatment with the dichloromethane-extracted fraction of graviola aerial part powder on apoptosis in the UW-BCC1 and A431 cell lines. Fluorescence microscopy was performed to evaluate apoptosis in (A) *UW-BCC1* and (B) *A431* cells treated with the dichloromethane (DCM) extracted fraction of graviola powder (0-10 µg/ml: 48h). Apoptotic cells were stained green with Annexin-V while necrotic cells stained red with propidium iodide. Details are described in Methods.
